# An atomic look at the interface of GHSR and its partners

**DOI:** 10.1016/j.csbj.2024.11.035

**Published:** 2024-11-22

**Authors:** Carlos A.V. Barreto, Irina S. Moreira

**Affiliations:** a PhD Programme in Experimental Biology and Biomedicine, Institute for Interdisciplinary Research (IIIUC), University of Coimbra, Casa Costa Alemão, Coimbra 3030–789 , Portugal; b CNC - Center for Neuroscience and Cell Biology, Center for Innovative Biomedicine and Biotechnology, University of Coimbra, Coimbra 3004–504, Portugal; c Department of Life Sciences, University of Coimbra, Calçada Martim de Freitas, Coimbra 3000–456, Portugal

**Keywords:** G protein-coupled receptor (GPCRs), Ghrelin receptor (GHSR), G protein, Arrestin, Selectivity

## Abstract

G protein-coupled receptors (GPCRs) regulate cellular activity by transducing external signals and selectively coupling them to intracellular partners. Ghrelin receptor (GHSR) has garnered significant interest over the past decade owing to its diverse functional roles. In this study, we simulated five distinct GHSR–partner complexes, including G_q_, G_i_, and arrestin in two conformational states, to investigate the structural determinants of partner coupling. Interface and contact analyses revealed conserved interaction sites and novel interactions that were specific to each partner family. Molecular dynamics simulations provided insights into GHSR conformational dynamics, highlighting notable differences in key structural regions across complexes, such as the TM5 bulge. Our findings underscore the structural diversity of GHSR coupling mechanisms and contribute to a deeper understanding of their functional versatility.

## Highlights

• Detailed conformational analysis of GHSR-partner complexes.
• Distinct conformational states and interaction interfaces for each partner complex.
• Common interaction sites for both G protein and arrestin (TM3, ICL2).
• Arrestin destabilises the receptor active conformation.

## Graphical Abstract

**Figure:**
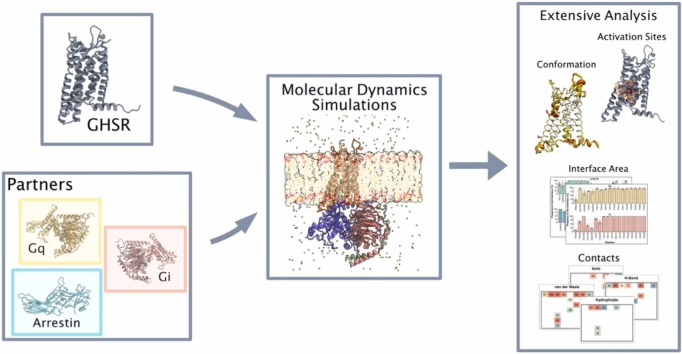

## 1 Introduction

G-protein coupled receptors (GPCRs) are the largest family of membrane proteins in the human body and are the targets of one-third of the drugs approved in the market [1]. This superfamily is responsible for communication between intracellular and extracellular environments. GPCRs change their conformation during receptor activation to accommodate intracellular partners (G proteins or arrestins) [2].

In the last few years, the increase in the number of experimental GPCR-partner structures available has revealed that most GPCR residues that interact with the intracellular partners are conserved, despite their low sequence identity [3], [4]. However, this does not explain how some GPCRs strongly bind to only one G protein subtype, whereas others seem to have a more promiscuous nature. Its important to note that the majority of this structural data is for complexes between class A GPCR and G protein, and partner interactions of other classes of GPCR or with different partner families (like arrestin) are still poorly understood. Unveiling the specific determinants of each class/partner subtype is essential to better understand and control GPCR signaling.

Ghrelin receptor (GHSR) is a promiscuous GPCR that has gained attention in the last decade. Long regarded as the ”hunger hormone”, more recent studies have shown its influence on neurological functions such as learning and memory [5], [6]. Furthermore, it has been proposed that these two functions (systemic and neurological) involve two distinct signalling pathways [7], [8]. In recent years, a few structures of active complexes of GHSR with different G protein subtypes have been solved experimentally [9], [10], [11], [12]. However, there are no studies that focus on comparisons in the dynamics of complexes that GHSR made with its partners.

Here, we present the results obtained from atomistic molecular dynamics simulations of GHSR complexes. We used three solved G protein complexes: one GHSR-G_q_ structure (primary coupling) with Ghrelin bound and two GHSR-G_i_ structures (secondary coupling), one bound to Ibutamoren and another with Ghrelin (hereinafter referred as G_q_, G_i_-Ibu and G_i_-Ghrl respectively). Additionally, we built models of GHSR-arrestin complexes based on two different GPCR-arrestin conformations: one based on the neurotensin receptor 1 (NTS_1_R)-arrestin complex (arr6UP7), and another based on the muscarinic 2 receptor (M_2_R)-arrestin complex (arr6U1N). We extensively characterised the conformation of GHSR and the interface with the different partners through all-atom molecular dynamics (MD) simulations.

## 2 Methods

### 2.1 Structures preprocessing

Two G protein-coupled conformations were extracted from the resolved structures. The G_q_-bound complex was extracted from PDBid 7F9Y [10], whereas the G_i_-bound complex (G_i_-Ibu) was retrieved from PDBid 7NA8 [11]. Refined versions of these structures were retrieved from GPCRdb [13], [14], and the receptor, ligand, and G protein structures were extracted to build the simulation systems. An additional G_i_-bound structure (G_i_-Ghrl) was extracted from PDBid 7NA7 [11] and used as an additional control for comparison between the G protein system results.

There are no resolved arrestin complexes for GHSR. Multiple orientations of nonvisual arrestin have been identified in class A GPCR. Two GHSR-arrestin complexes were constructed to investigate the two most extreme orientations, i.e, an orientation similar to that of the rhodopsin-visual arrestin complex and an alternative arrestin orientation that was the most different from the rhodopsin one. Templates were chosen based on the highest sequence similarity between GHSR and receptors that have available arrestin complexes resolved. For the Rhodopsin-like one, the M_2_R-arrestin-2 complex (PDBid: 6U1N [15]) was used (sequence similarity to GHSR of 26 %), while the complex NTS_1_R-arrestin-2 (PDBid: 6UP7 [16]) was used for the alternative orientation (sequence similarity to GHSR of 40 %). The receptor-ligand conformation was retrieved from the G_q_ complex (PDBid: 7F9Y [10]). Arrestin-3 structures were modelled using the MODELLER package [17], [18]. Two arrestin models were built using each template with Uniprot [19] sequence ID P49407. The final complexes were built by superimposition of the GHSR-ghrelin conformation and arrestin-3 models with resolved arrestin complex structures (PDBid: 6U1N [15] and 6UP7 [16]).

### 2.2 Molecular dynamics simulations

#### 2.2.1 Membrane orientation

The orientation of the membrane was retrieved from the Orientations of Proteins in Membrane (OPM) database for every structure [20]. The orientation of the resolved arrestin structures was used for the arrestin complexes. The receptor structures were aligned with the OPM-retrieved structures from TM1 to TM4.

#### 2.2.2 System building

The oriented complexes were inserted into a bilayer lipid membrane in a cubic simulation box hydrated with TIP3P and 0.15 M NaCl using the CHARMM-GUI webserver [21], [22], [23]. The ACE/CT1 termini were used as the N- and C-termini. Disulfide bonds were defined as reported in UniProt for each protein [19]. A heterogeneous lipid bilayer was built around the receptor structure with 1-palmitoyl-2-oleoyl-sn-glycero-3-phosphocholine (POPC) and cholesterol (CHL1) (ratio 9:1), with 200 lipids per leaflet. Water was added to the box with hydration of 150 (150 water molecules per lipid). The final complexes had between 220 and 250 thousand atoms.

#### 2.2.3 Simulation parameters

MD simulations were performed using the GROMACS 2022.5 [24] and CHARMM36m force field [25]. The systems were equilibrated and simulated using an NPT ensemble. During initialization, to achieve and maintain the desired temperature (310 K), a V-rescale thermostat was used with a coupling constant of 0.1 ps. Pressure coupling was performed using a semi-isotropic Berendsen barostat at 1 bar with a compressibility of 4.5 × 10^−5^ bar and a coupling constant of 1.0 ps. For the production phase, the thermostat was switched to Nosé -Hoover, and the barostat was switched to Parrinello-Rahman. Electrostatic interactions were computed with the Particle-Mesh Ewald (PME) method [26], with a Fourier grid of 0.12 nm and a cutoff of 1.2 nm for direct contributions. Lennard-Jones interactions were computed using a non-bonded neighbor pair list with a cutoff of 1.20 nm, enabling the Verlet scheme. Solute bonds were constrained using a Parallel LINear Constraint Solver (p-LINCS) [27]. The steepest descent algorithm was used to minimize the initial energy of the system through a 50000-step run. The systems were then initialized for 26 ns with sequential runs of 2 ns each with successively lower force constraints on the heavy lipid and protein atoms. A final initialization step of 5 ns was performed without any constraint forces. Triplets of 1000 ns were produced for each complex, except for the G_i_-Ghrl system, in which each replicate was 500 ns, resulting in a total simulation time of 13.5 μs.

### 2.3 Analyses

#### 2.3.1 Equilibration analysis

To determine when the system was well equilibrated, we examined six different measures: i) helicity of the receptor structure, ii) *z*-component of the distance between the centre of mass of the membrane and the centre of mass of the receptor, iii) box area, calculated as the product of *x* and *y* of the simulation box, iv) root-mean-square deviation (RMSD) of Cα from the transmembrane region of GHSR using the initial frame of the system, v) RMSD of Cα from the partner structure using the initial frame of the system vi) RMSF values of GHSR, G proteins and arrestins. The sections of simulation time defined as non-equilibrated were discarded for the remainder of the analysis.

#### 2.3.2 Conformational analysis

The four systems used metrics to assess the GPCR activation state and compare the receptor conformation. The following calculations were performed: i) the TM3-TM6 distance measured between Cα atoms of Arg141^3.50^ and Val262^6.34^, ii) the TM3-TM7 distances measured between Cα of Arg141^3.50^ and Tyr323^7.53^, and between the center of mass of side chains of Ile134^3.43^ and Tyr323^7.53^, iii) RMSD of the NPxxY motif backbone relative to the inactive structure, iv) transmission switch changes were analyzed using Val131^3.40^ – Trp276^6.48^ and Val225^5.51^ – Phe272^6.44^ distances, v) Na^+^ pocket area, by calculating the distances between the center of mass of side chains of Asp89^2.50^, Thr130^3.39^, and Asn319^7.49^, vi) hydrophobic lock area, by calculating the distances between the center of mass of side chains of Ile134^3.43^, Val268^6.40^, and Val269^6.41^, vii) distance between the Cα of Asp89^2.50^ and the closest Na^+^ ion, and viii) number of water molecules within 8 Å of Asp89^2.50^. All analyses were performed using the GROMACS 2022.5 tools, and the Ballesteros-Weinstein enumeration scheme [28].

#### 2.3.3 Ensemble analysis

The ensemble analysis was performed using the PENSA Python package [29]. The backbone and side-chain torsion distributions during the simulations were compared using the Jensen-Shannon distance (JSD). Six independent comparisons were performed: GHSR-G_q_ vs. GHSR-G_i_-Ibu, GHSR-G_q_ vs. GHSR-G_i_-Ghrl, GHSR-G_i_-Ibu vs. GHSR-G_i_-Ghrl, GHSR-arrestin (6U1N) vs. GHSR-arrestin (6UP7), GHSR-G_q_ vs. GHSR-arrestin (6U1N), and GHSR-G_q_ vs. GHSR-arrestin (6UP7).

#### 2.3.4 Interface area

The interfacial area was calculated from the Solvent-Accessible Surface Area (SASA). The SASA was calculated for every residue in the complex throughout the simulation. The interfacial area is calculated as follows:

$$\mathrm{Interface}\;\mathrm{Area}=\;\frac{{∆\mathrm{SASA}}_{\mathrm{GHSR}}+{∆\mathrm{SASA}}_{\mathrm{PARTNER}}}{2}$$

where ∆SASA is the difference between the SASA of the unbound (SASA_mon_) and bound (SASA_complex_) structures.

To study the interface behavior over time, we applied our previously described protocol [30]. First, we assessed the residues at the beginning of the simulations by calculating ΔSASA as the difference between SASA in the monomer and SASA in the complex. Residues were classified as part of the interface if the ∆SASA was higher than 0. To reduce the list of residues that had ΔSASA lower than 0.05 nm^2^ and a normalized ΔSASA lower than 10 % were excluded. The ΔSASA was normalized by dividing the SASA value of each residue by the SASA_max_ of that type of residue. The presence of these original interface residues was analysed throughout the simulation, as well as any additional residues whose presence at the interface was higher than 50 %.

#### 2.3.5 Interactions

Interactions between GHSR structures and partners were assessed using the getcontacts Python package. The interactions were grouped into five different categories: i) ionic, ii) salt bridges, iii) aromatic, contains all π-cation, π-stacking, and T-stacking, iv) H-bonds, contains all hydrogen bonds (backbone-backbone, backbone-side chain, and side chain–side chain), v) hydrophobic interactions, and vi) van der Waals interactions. The occupancy of each interaction was calculated for each replica and averaged for each system. Interactions with an average occupancy of less than 30 % were excluded.

## 3 Results & discussion

All-atom simulations of the five GHSR partner complexes were conducted to study the specific determinants of GHSR for each partner. The complex simulations were replicated three times, each for 1000 ns, except for the G_i_-Ghrl system, in which each replicate was 500 ns, for a total duration of 13.50 μs.

### 3.1 Equilibration of the systems

Various metrics were assessed to determine the equilbrated sections of the simulations, including the helicity of the GHSR, distance between the centre of mass of the receptor and the average position of the membrane P atoms, and *xy* area of the simulation box. Based on these metrics, the first 200 ns of each simulation were classified as non-equilibrated period and excluded from the subsequent analysis (Fig. S1-S3). RMSD and RMSF of receptor (only transmembrane region) and partners was also assessed to determine the stability of the complex. The partners had higher RMSD than receptors, which was expected because the G protein and arrestin are soluble proteins. Nevertheless, the structures were stable over time, with no major movements after the initial 200 ns (Fig. S4-S8).

### 3.2 Conformational analysis

3.2

**Fig. 1:**
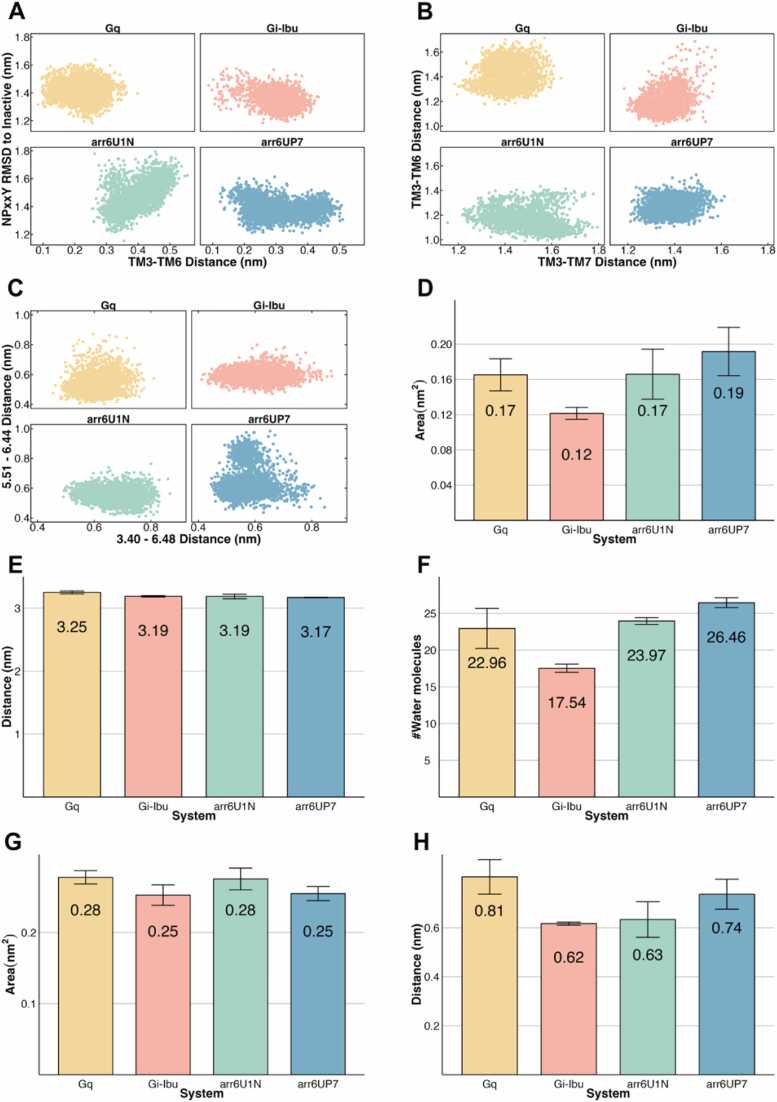
**:** Analysis of GPCR activation sites in different simulated systems. A - Comparison of the TM3-TM6 distance (measured between Cα of Arg141^3.50^ and Val262^6.34^) and the RMSD of NPxxY motif’s backbone to the inactive structure; B - Comparison of the TM3-TM6 distance (measured between Cα of Arg141^3.50^ and Val262^6.34^) and the TM3-TM7 distance (measured between Cα of Arg141^3.50^ and Tyr323^7.53^); C - Comparison of distances within Transmission Switch, represented as the distances between Val131^3.40^ and Trp276^6.48^ side chains and Val225^5.51^ and Phe272^6.44^ side chains; D - Na^+^ pocket area, calculated using the triangle of distances between Asp89^2.50^, Thr130^3.39^ and Asn319^7.49^; E - Distance between Asp89^2.50^ and the closest Na^+^ ion; F - Number of water molecules within 8 Å of residue Asp89^2.50^; G - Hydrophobic Lock area, calculated using the triangle of distances between Ile134^3.43^, Val268^6.40^, and Val269^6.41^; H - Distance between side chains of Ile134^3.43^ and Tyr323^7.53^. The bars represent the mean ± SEM of each system. The systems are colour-coded as G_q_ - yellow, G_i_ - red, arr6U1N - green, and arr6UP7 - blue.

GPCR activation metrics were used to investigate the differences between conformations bound to different partners. These metrics have been previously applied to distinguish different activation states from inactive to intermediate to active. First, we compared the distance between the Cα atoms of residues Arg141^3.50^ and Val262^6.34^. This measure evaluates the outwards movement of TM6 that occurs as a result of GPCR activation. RMSD of the NPxxY region to the inactive state and distance of the Cα atoms of Arg141^3.50^ and Tyr323^7.53^ was then evaluated and plotted against the TM3-TM6 distance (Fig. 1A-B). The former evaluates the conformation of an important motif from TM7 that kinks during activation, while the latter evaluates the packing between TM3-TM7, which becomes tighter with activation. TM3-TM6 distance when plotted against RMSD values of the NPxxY region to the inactive state showed a higher TM3-TM6 distance and lower NPxxY RMSD on the G_q_-bound system when compared to the other G protein complexes. Interestingly, the M_2_R-derived arrestin conformation showed the largest difference in the NPxxY motif compared to the inactive state of GHSR. Looking at the distance of TM3-TM7, the two G protein-bound complexes as well as the arr6UP7 complex are clustered around the 1.4 nm mark, while the arr6U1N complex seems much more dynamic, reaching higher distances more consistently.

Next, we assessed the conformation of the transmission switch region, a motif regarded as the initial step of GPCR activation, by calculating two pairwise distances between residues Val131^3.40^ and Trp276^6.48^, and between residues Val225^5.51^ and Phe272^6.44^. The interaction between these two pairs of residues is linked to the active state. The four systems showed distances higher than 0.4 nm in both pairs, with average values between 0.50 and 0.70 nm (Fig. 1C). The G_q_-bound system showed the lowest distances between Val225^5.51^ and Phe272^6.44^, while arr6UP7 showed distances higher than 0.8 nm. For the other pair, Val131^3.40^– Trp276^6.48^, distance values were even more dispersed throughout the simulation, but again G_q_-bound showed the lowest variation out of the four systems. This suggests that the conformation of the transmission switch region is more stable in the G_q_-bound system, whereas for the other three systems, this region is quite flexible.

As a third metric, we used the Na^+^ pocket area, calculated as a triangle of distances between TM2-TM3-TM7. This pocket, usually filled with water and a sodium ion in the inactive state, collapses during activation, eventually leading to the rewiring of NPxxY contacts. The G_i_-Ibu complex had a smaller area than the other systems (Fig. 1D), with an average area of 0.12 nm^2^. We also analysed the presence of Na^+^ ions and water molecules in the core of the receptor. Across all the systems, no Na^+^ ions approached the Na^+^ pocket, with average distances exceeding 3 nm (Fig. 1E). Furthermore, when analysing the number of water molecules within an 8 Å radius of Asp89^2.50^, we found that arrestin-bound complexes consistently exhibited a higher number of water molecules than the other systems, whereas the G_i_-Ibu system showed fewer water molecules (Fig. 1F). For the G_q_-bound system, we found discrepancies between replicas; one replica exhibited an average water count similar to that of the G_i_ systems, while another showed counts closer to the arrestin-bound systems. These variations in the water molecule distribution near the Na^+^ pocket may reflect differences in receptor dynamics, with arrestin-bound systems potentially representing an intermediate or preparatory state toward receptor deactivation. While the G_i_ systems exhibited a smaller pocket area, consistent with activation, the higher water molecule density observed in other systems, particularly in arrestin-bound complexes, suggests subtle differences in receptor conformations that could be linked to receptor functional states. These results are consistent with the general trends observed for GPCRs. However, given the observed variability across replicas, these findings should be interpreted with caution and framed as part of the broader conformational landscape of the GHSR.

Similar to the Na^+^ pocket, we calculated the hydrophobic lock area with distance values of Ile134^3.43^, Val268^6.40^, and Val269^6.41^. This region of the receptor is a cluster of hydrophobic residues (hence, the name) that stabilizes TM3 and TM6 in an inactive state, and is disrupted upon receptor activation. All systems showed an area larger than 0.2 nm^2^ (Fig. 1G), and no significant differences were found between the systems.

Lastly, the TM3-TM7 packing was again assessed, but now with a distance between Ile134^3.43^ and Tyr323^7.53^ side chains. This is an important interaction for the active state of GPCRs, as it helps stabilize the TM3-TM7 interaction. Upon activation, the hydrophobic lock is disrupted and residue Ile134^3.43^ shifts its interactions from Val268^6.40^ and Val269^6.41^ to Tyr323^7.53^. Here, the G_q_-bound system showed the highest value, followed by the arrestin complexes and the G_i_-bound structure, suggesting looser TM3-TM7 packing in these systems.

The G_i_-Ghrl system showed results closer to those observed in the G_i_–Ibu system, suggesting that the conformational changes in GHSR seen between the two G protein systems are due to two different couplings in the intracellular pocket and do not come from differences in the ligand (Fig. S9).

### 3.3 Ensemble analysis

**Fig. 2:**
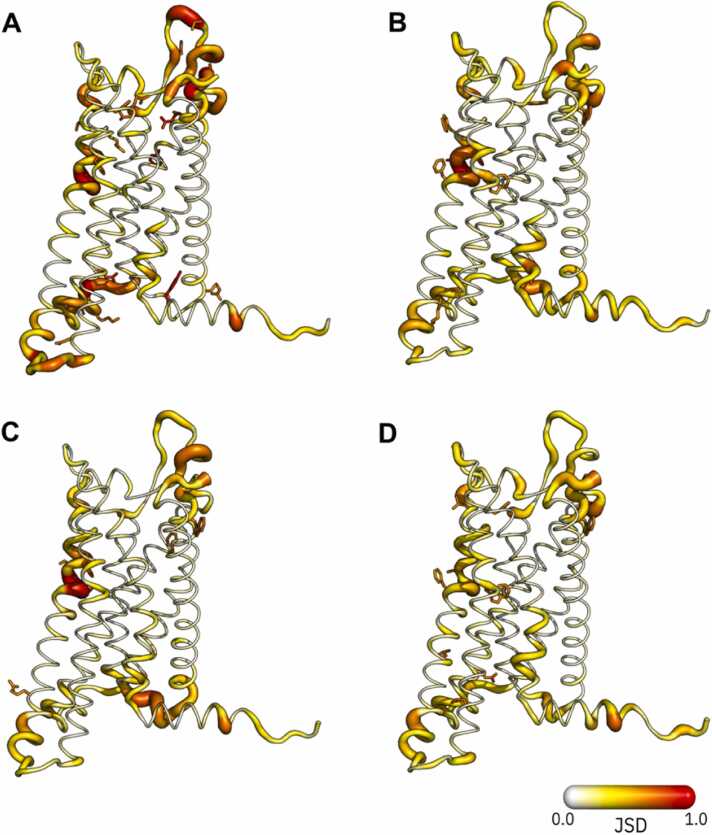
**:** Comparison of torsion angles of backbone and side chain via JSD. A. Comparison of G_q_-bound and G_i_-Ibu systems. B - Comparison between the two arrestin systems. C - Comparison between the G_q_-bound system and arr6U1N. D - Comparison between G_q_-bound system and arr6UP7. The backbone and side chains were coloured according to the JSD values from white (0.0) to red (1.0). Side chains are shown for residues with a JSD higher than 0.6. The structures were visualised using PyMol software [32].

Ensemble analysis was performed using the PENSA Python package to obtain an overview of the differences between the receptor conformations of the five complexes. Here, we used the JSD of the distribution of backbone and side-chain torsions to compare receptor conformations. Firstly, four separate comparisons were performed: G_q_ vs. G_i_-Ibu, arr6U1N vs. arr6UP7, G_q_ vs. arr6U1N, and G_q_ vs. arr6UP7 (Fig. 2). Two additional comparisons were performed (G_q_ vs. G_i_-Ghrl and G _i_-Ibu vs. G_i_-Ghrl) (Fig. S10).

Of all the comparisons, the three G protein comparisons generated high JSD values (> 0.6) for a larger number of residues, both in the backbone and side chain torsions. Comparing G_q_ to G_i_-Ibu, some of these residues are located in ECL2 and the adjacent ends of TM4 and TM5. This may be due to the difference in ligands between these two complexes, as ghrelin is much larger than ibutamoren. Indeed, when comparing G_q_ with the G_i_-Ghrl system, the differences in ECL2 are less pronounced. Additionally, a comparison between the two G_i_ systems shows mainly differences in the ECL2. At the intracellular end, comparison of G_q_ to the two G_i_ systems show the highest JSD values for backbone torsions are at the end of TM3 (Ile145^3.54^) and most of the ICL2 (residue Val154^34.56^ with the highest JSD value), suggesting that this substructure is a key difference between the two G protein families (Fig. S11). Note that this ICL2 difference is more pronounced between G_q_ and G_i_-Ghrl despite having the same ligand. To a lesser extent, ICL3, TM6 and H8 also exhibited differences in the backbones between G_q_ and G_i_-bound systems (Fig. S11). Looking for the side chain torsions at the intercellular side, we observed maximum differences of Glu140^3.49^ and Tyr330^8.50^ between the G_q_-bound and G_i_-Ibu complexes. Additionally, a comparison between G_i_-Ghrl and the two other G protein systems showed differences in the intracellular ends of TM5 and TM6 (Fig. S12).

When comparing the two arrestin conformations, we did not observe massive changes in the backbone or side-chain torsion distribution. ICL1 (Arg75^12.51^), TM2 (Thr76^2.37^), TM7 (Asn324^7.54^), and H8 (Tyr330^8.50^) showed the highest JSD when examining the backbone torsion distribution on the intracellular side of the receptors, whereas residues Asn79^2.40^, Ile145^3.54^, and Arg243^5.69^ showed the highest JSD for side chain torsion (Fig. S11-S12).

Similarly, the G_q_-bound and arrestin conformations did not show significant differences at the intracellular site. Backbone torsion showed differences in ICL1 and TM2 when comparing G_q_-bound arr6U1N, whereas arr6UP7 showed a more identical distribution in these substructures (Fig. S11-S12).

Importantly, all comparisons, except for the comparison between the two G_i_-bound systems, showed high JSD values (backbone and side chain) in a section of TM5 known as the TM5 bulge (residues Ile219^5.45^, Phe220^5.46^, and Phe221^5.47^) (Fig. 2, Fig. S10-S12). This region of the receptor has been linked to some of the most important activation motifs, such as PIF, the Na^+^ pocket, and the NPxxY motif, and might indicate changes in the stability of the receptor conformation, especially when we compare the G_q_-bound arrestin complexes [31].

### 3.4 Interface area

**Fig. 3:**
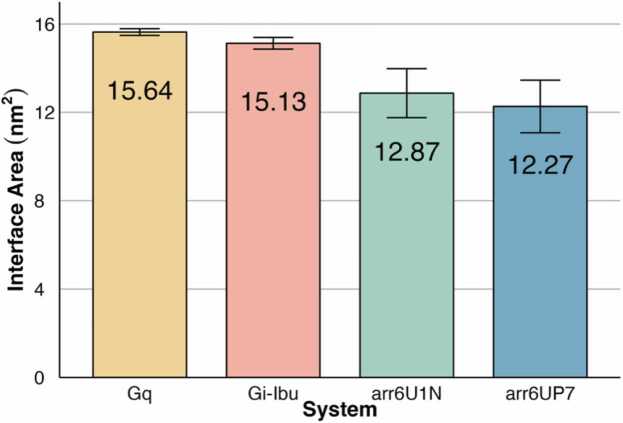
**:** Interface area of GHSR - partner systems. Replicates are summarized as the mean ± standard error of the mean. Systems are colour-coded: G_q_, yellow; G_i_-Ibu, red; arr6U1N, green; and arr6UP7, blue.

The interface areas of the complexes were studied using SASA calculations to assess the differences between the complexes (see Methods section for more information). The interface areas of GHSR, G proteins, and arrestins were consistent over time across all replicates (Fig. S13–S15), indicating that all four complexes were stable and that there was no decoupling in any replica. The average total interface area was higher for G protein complexes than for arrestin complexes (Fig. 3). The G_q_-bound complex showed the widest average area of interface (15.64 ± 0.15 nm^2^) of all the complexes simulated. The G_i_-Ibu showed the second-highest average area (15.13 ± 0.27 nm^2^). The G_i_-Ghrl system showed values similar to those of the G_i_-Ibu system (14.18 ± 0.78 nm^2^) (Fig. S16). The arrestin complexes showed 12.88 ± 1.19 nm^2^ and 12.27 ± 1.12 nm^2^ for the complex with the classic orientation and the alternative orientation, respectively (Fig. 3). This is consistent with the differences reported between G protein and arrestin interfaces, where arrestin, although it usually interacts with more regions of the receptor, the interface is divided into patches, whereas G protein interfaces tend to be more contiguous [33].

The contribution per protein to the interface area showed that the difference between the G protein and arrestin complexes originates from the GHSR area. Again, the highest area was in the G_q_-bound structure (15.77 ± 0.28 nm^2^) and the second highest area was in the G_i_-bound structure (G_i_-Ibu: 15.44 ± 0.28 nm^2^, G_i_-Ghrl: 15.27 ± 0.89 nm^2^) (Fig. S17). The GHSR on the arrestin complexes showed an average interface area of 12.79 ± 1.07 nm^2^ for the M2-derived orientation and 11.81 ± 1.23 nm^2^ for the NTS1-derived orientation. These arrestin values are similar to those reported in the literature for rhodopsin and β_1_ adrenergic receptor (β_1_AR) arrestin complexes [34], [35]. The G protein interface area, although higher than that of arrestin complexes, is much smaller than the 25 nm^2^ reported for the β_2_ adrenergic receptor (β_2_AR)-Gs complex [35], [36]. When comparing the contributions of the Gα subunit and arrestins, both showed equivalent average areas at the interface. However, the G_q_-bound structure shows a slightly higher interface area of the Gα subunit than the G_i_-bound structures (G_q_ = 13.31 ± 0.41 nm^2^; G_i_-Ibu = 11.63 ± 0.21 nm^2^; G_i_-Ghrl = 12.06 ± 0.37 nm^2^). The Gβ subunit of the G protein was also present in the interface, but in this case is the G_i_ system shows higher interface area than the G_q_ system.

A detailed analysis of the area of the interface at the substructure level revealed specific behaviors between and within each class of partners. For GHSR, TM6 and ICL2 were the two major substructures involved in the interface between the receptor and partner (Fig. S18). TM6 showed a higher contribution in G protein complexes compared to arrestin complexes, whereas ICL2 showed equivalent area values for all complexes, except for the arr6UP7 complex. Both TM3 and TM5 showed higher average areas in the arr6U1N complex than in the other three structures. H8 showed a much larger area of the arr6UP7 complex, followed by the G_i_-Ghrl system. Looking at the G protein side, more specifically the Gα subunit, as expected, the majority of the interface was concentrated in the H5 substructure, with equivalent areas in all G protein systems, although slightly higher for the G_q_-bound complex (Fig. S18). The contribution of the rest of the substructures present at the interface is minimal compared to H5 and the equivalent between the systems. On the arrestin side of the interface, loop s5s6, commonly known as the finger loop, showed the highest contribution to the interface of the complex (Fig. S18). For the NTS_1_R-derived arrestin conformation loops, s8s9 (middle loop) and s17s18 (lariat loop) showed slightly higher interface areas than the M_2_R-derived conformation, whereas the latter showed higher interface areas on the s15s16 loop (C-loop).

Sandhu et al. and Matic et al. recently published extensive studies on GPCR-G protein interfaces[3], [4]. While the first simulated six different complexes, the latter used 362 different structures with no dynamic data. Both reported ICL2 and TM6 as substructures with more contact with the G protein and H5 with the most contact with the receptors in both G_i_ and G_q_ complexes. Sandhu et al. also showed that G_q_ has a more specific contact with receptors through hns1, which might explain the higher interface area observed in this substructure in the G_q_-bound complex than in the G_i_-bound complexes [3]. Furthermore, the differences found between the two arrestin complexes, especially at the receptor side of the interface, are in agreement with those reported by Huang et al.[16] and our previous studies on dopamine and opioid families[37], [38]. The 90º rotation of arrestin changes its interactions with the receptor, but mostly on the receptor side, whereas the interacting motifs of arrestin remain similar. One major change observed here was the higher interface area of H8 with the arr6UP7 system compared with that of the M_2_R-derived conformation. This substructure has been reported to be crucial for the binding of visual arrestin to activated rhodopsin [34], [39].

**Fig. 4:**
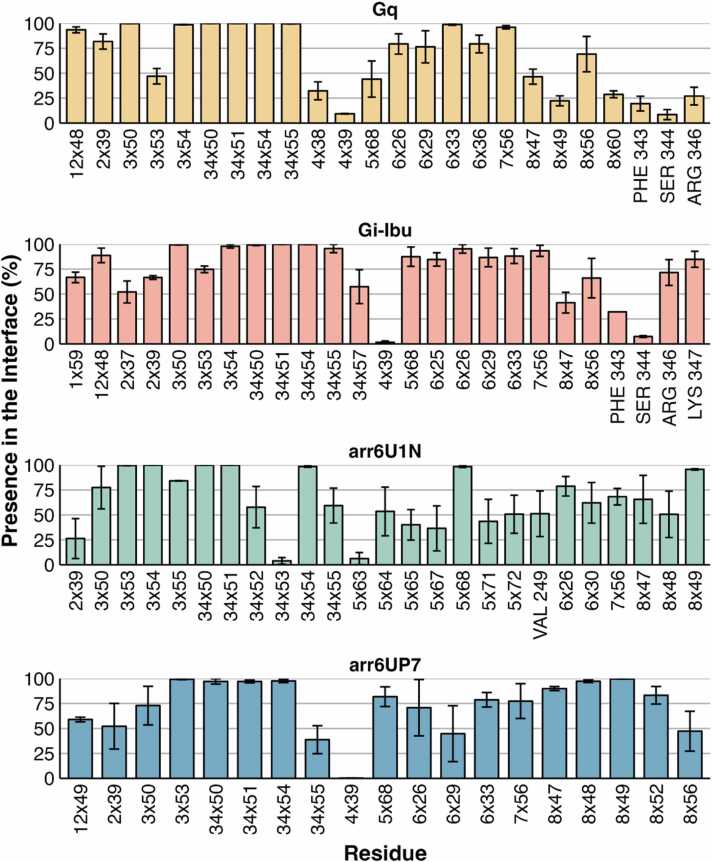
**:** Original decoy interfacial residues for GHSR. Replicates are summarized as the mean ± standard error of the mean.

Looking at the receptor side of the interface, most residues of the original interface continued to be part of the interface for more than half of the simulation time, with some exceptions (Fig. 4 and S19). Various residues were common between all interfaces, especially those at the intracellular ends of TM3 and ICL2, such as Arg141^3.50^, Pro148^34.50^, Leu149^34.51^, and Lys152^34.54^. Residue Thr78^2.39^ also shows in all initial interfaces, although it has no presence in the arrestin systems simulations. Regarding the differences between the G protein systems, Arg70^1.59^, Thr76^2.37^, and Leu253^6.25^ were only present in the G_i_ interfaces, whereas Met264^6.36^ and Phe340^8.60^ were only present in the G_q_-bound system. Residues that were not part of the original interface but consistently present at the interface are shown in Fig. S22. G_q_-bound system additional presence of ICL3 residues, such as Val248, Val249, and Gly250. These residues were also present in both G_i_-bound systems, but in less than 50 % of the cases. Residue Leu253^6.25^, which is part of the original interface in the G_i_-Ibu system, was shown to be an additional residue for G_q_-binding. Furthermore, residues Val155^34.57^ and Arg242^5.68^ were not part of the original interface of the G_i_-Ghrl system, but showed very high occupancy at the additional interface, making them specific to the G_i_ interfaces. For arrestin-bound structures, arr6U1N was distinguished by the presence of an extended set of residues in TM5, where the only common residue at the arr6UP7 interface was Arg242^5.68^ (Fig. 4). As for additional residues, in arr6U1N, only two additional residues showed a presence higher than 50 %, one in TM3 (Phe147^3.56^) and TM6 (Thr261^6.33^), whereas for the arr6UP7 system, residues Thr76^2.37^, Ile145^3.54,^ and Lys347 showed a presence higher than 50 % (Fig. S22).

On the G protein side of the interface, almost all residues identified at the original interface had high presence values, except for some residues in HN and s2s3 (Fig. S20). Only a few additional residues were identified as part of the interface, such as Ser320^h4s6.11^ and Thr321^S6.03^ in the G_q_-bound system, Glu318^h4s6.12^ in the G_i_-Ibu system and Lys349^H5.21^ in the G_i_-Ghrl system (Fig. S23).

In arrestins, the residues of the finger loop identified at the initial interface had the highest values of presence throughout the simulation (Fig. S22). Although C-loop residues are part of both initial interfaces, their presence is higher in the arr6U1N system. For additional residues, Gly65^s5s6.01^ and Arg66^s5s6.02^ were detected in the arr6U1N system, whereas Asp70^s5s6.06^ and Asp136^s8s9.06^ were consistent in arr6UP7 (Fig. S24).

### 3.5 Key molecular interactions

**Fig. 5:**
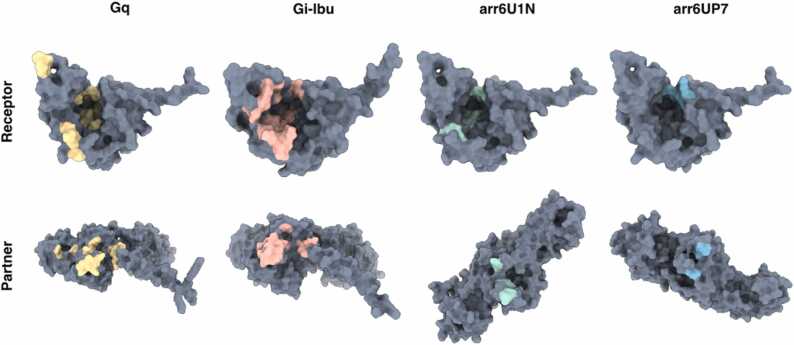
**:** Most prevalent interactions for each system. Receptors and partners are represented as surfaces, and interacting residues are colored.

The *getcontacts* package was used to study specific interactions between the receptor and partners. Most of the contacts observed were weak hydrophobic interactions, with the exception of the G_i_ complexes. Fig. 5 shows the contacting residues with occupancy greater than 50 %, excluding the interaction type. G protein complexes show a more continuous area of interactions on the receptor side, whereas arrestin contacts in GHSR appear sparser. This is especially evident in the deeper region of the intracellular pocket of GHSR, where only the G protein interacts more consistently, particularly G_i_. This result was in agreement with the differences observed in other class A GPCRs. The patchy nature of the arrestin interface has been suggested to help bind to a more diverse set of receptors, whereas G protein-binding is usually more selective [3], [33].

More detailed contact matrices divided by the interaction type are shown in Figs. S25–S34. GHSR and both G proteins mainly establish interactions through TM2, TM3, ICL2, TM6, and TM7 on the receptor and H5 on the intracellular partner. This was somewhat expected, as G_i_ and G_q_ complexes tend to show more similarities, especially when compared to G_s_ complexes [3]. Interestingly, both G_i_-bound systems also showed interactions between TM5 and H5, which was not observed in the G_q_-bound system. The interactions in the G_q_-bound system were mainly vdW, with one aromatic interaction between TM3 and H5. In contrast, both G_i_ systems established stronger interactions (ionic and H-bonding) with the GHSR. This might explain why receptors that primarily bind G_q_ tend to have a more promiscuous nature, as G_q_ interactions are weaker.

Ionic interactions were formed between Asp341^H5.13^ of G_i_ and Arg242^5.68^ and Arg254^6.26^ on the receptor. A third ionic interaction between Glu318^h4s6.12^ and Arg254^6.26^ was also identified in both G_i_ complexes. Pairs 5.68 – H5.13, previously identified by Sandhu et al., have a specific G_i_ contact, whereas the other two seem to be novel or specific to GHSR [3]. Additionally, G_q_ has interactions between ICL3 and both H4 and S6, whereas G_i_ systems seem to shift to the closest substructures, that is, TM6 and h4s6, respectively. Common contacts were observed among the three G protein complexes: 3.50 - H5.23, 34.50 - H5.16, and 7.56 - H5.24. The latter was described as a G_i_-specific contact, whereas GHSR showed no preference between the two G proteins. Additionally, the contact between 3.53 and H5.19 was identified as common between the G_i_ and G_q_ interfaces was observed in all three G protein complexes, although with significantly less occupancy in the G_q_-bound system [3], [4]. Interaction between 34.55 - hns1.02, previously identified as a G_q_-specific contact, is present in all G protein systems, although it has significantly less occupancy on the G_i_-bound systems.

The arr6U1N system was the only arrestin system that showed a high occupancy (50 %) of H-bonds or ionic interactions between the receptor and partner, specifically between the H8 and s5s6 loops. Both arrestin systems showed interactions between TM3, TM7 and H8 and the s5s6 loop. Additionally, the arr6U1N system showed interactions between ICL2 and TM6, and the s5s6 loop. This is an expected behaviour observed by Yin et al. and from our previous studies in the Dopamine and Opioid family [37], [38].

For arrestins, most of the high-occupancy interactions are vdW interactions, although the interaction substructures differ between the two systems. There is no interaction with an occupancy higher than 50 %, which is common between the two systems. Furthermore, the NTS_1_R-derived system tended to show interactions with higher occupancy than the M_2_R-derived system, such as s5s6.07–7.56 and s5s6.05–8.47, with occupancies of 74 % and 89 %, respectively.

## 4 Conclusions

Understanding the determinants of the interaction between GPCR and their intracellular partners is one of the most challenging and studied problems in the GPCR field. Knowledge of specific interactions or conformations might unlock new therapeutics, especially when different signalling pathways are linked to distinct functions, such as GHSR. In this study, we simulated and analysed five GHSR complexes in detail. We identified differences in receptor conformation induced by different intracellular partners, especially arrestins. Additionally, we were able to structurally and dynamically characterise all four interfaces and identify common and specific patterns and interactions of all complexes. The G_q_-bound complex showed a greater TM3-TM6 distance and lower NPxxY RMSD, indicating a more active state relative to the G_i_-bound systems. Arrestin complexes, particularly arr6UP7, exhibit a distinct NPxxY motif, suggesting a unique conformational state that differs from both the G protein-bound and inactive states.

Analysis of the transmission switch region revealed that the G_q_-bound system maintained more stable pairwise distances between residues critical for activation, whereas arrestin complexes displayed greater flexibility. The Na^+^ pocket region, which typically collapses during GPCR activation, was the smallest among the G_i_-bound complexes, indicating a more active state. The G_q_-bound complex had the largest interface area, followed by the G_i_-bound and arrestin complexes, suggesting a more extensive interaction surface with G proteins, consistent with previous observations that G protein interfaces are more contiguous. Key molecular interactions revealed that G protein interactions with GHSR are more continuous and involve TM3, ICL2, and TM6, primarily through van der Waals and hydrophobic interactions. Notably, G_i_ interactions include stronger ionic and hydrogen bonds, which explain the specificity and strength of G_i_ binding compared to G_q_. Ensemble analysis of the torsion angle distributions revealed notable dissimilarities between the G protein and arrestin complexes. The high JSD values observed in the backbone and side-chain torsions of TM4, TM5, and intracellular loops highlight the structural adjustments unique to each complex. Notably, the TM5 bulge, which is critical for activation motifs, exhibited high JSD values across all comparisons, emphasising its role in maintaining the stability of the receptor conformation.

The distinct conformational states and interaction interfaces emphasise the intricacy of GPCR activation and deactivation mechanisms. The complexity of these interactions greatly increases when we start to consider other modulating factors such as different lipid membrane composition (in particular with PIP2), which was not studied in this work [40], [41]. Understanding these dynamics is vital for designing drugs that can specifically target particular pathways and offer potential therapeutic advantages for conditions regulated by GHSR.

## Funding

This work was supported by the European Regional Development Fund through the COMPETE 2020–Operational Programme for Competitiveness and Internationalisation, and Portuguese National Funds via Fundaçao para a Ciência e a Tecnologia (FCT) [LA/P/0058/2020, UIDB/04539/2020, UIDP/04539/2020, and DSAIPA/DS/0118/2020, http://doi.org/10.54499/DSAIPA/DS/0118/2020). C.A.V.B. was supported by the 10.13039/100006129FCT through a PhD scholarship SFRH/BD/145457/2019.

## CRediT authorship contribution statement

**Irina S. Moreira:** Writing – review & editing, Supervision, Resources, Project administration, Funding acquisition, Conceptualization. **Carlos A.V. Barreto:** Writing – review & editing, Writing – original draft, Visualization, Validation, Software, Methodology, Formal analysis, Data curation.

## Declaration of Competing Interest

None.
