# Supplementary material for: An atomic look at the interface of GHSR and its partners
Source: Comput Struct Biotechnol J. 2024 Nov 22;23:4242–51. doi: 10.1016/j.csbj.2024.11.035 (PMC11629268; doi:10.1016/j.csbj.2024.11.035)
Supplement: Supplementary file 1 — Supplementary material [file mmc1.docx]

**Supporting Information**

**An atomic look at the interface of GHSR and its partners**

Carlos A.V. Barreto†,‡ and Irina S. Moreira∗,‡,¶

†PhD Programme in Experimental Biology and Biomedicine, Institute for Interdisciplinary Research (IIIUC), University of Coimbra, Casa Costa Alemão, 3030-789 Coimbra, Portugal

‡CNC - Center for Neuroscience and Cell Biology, Center for Innovative Biomedicine and Biotechnology, University of Coimbra, 3004-504 Coimbra, Portugal

¶Department of Life Sciences, University of Coimbra, Calçada Martim de Freitas, 3000-456 Coimbra, Portugal


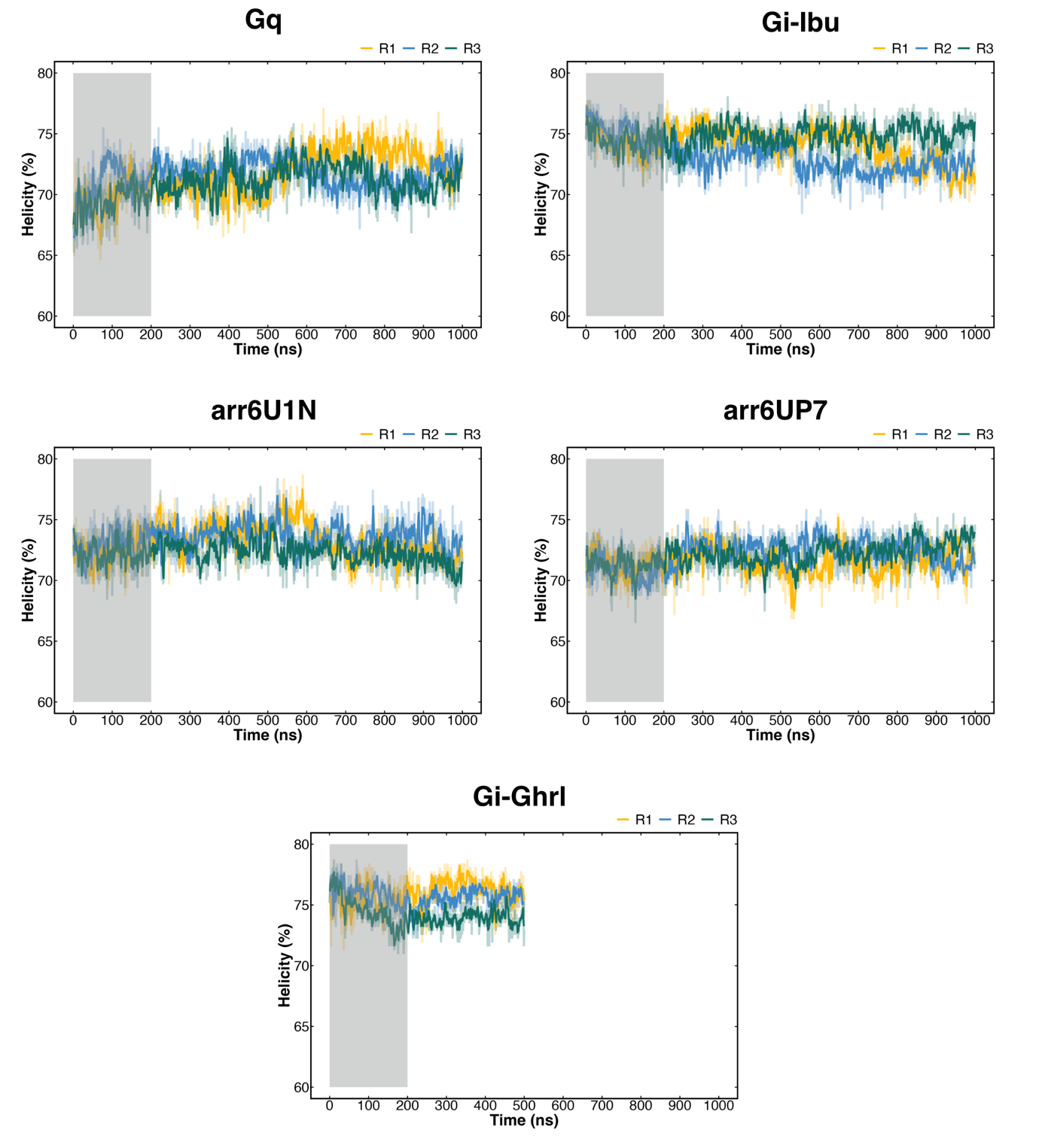


**Figure S1:** **Helicity (percentage of residues in the α-helix) of GHSR throughout MD simulations.** Replicas are colour-coded as follows: R1, yellow; R2, blue; and R3, green. The gray area marks the first 200 ns and is excluded from the remaining analysis.

**
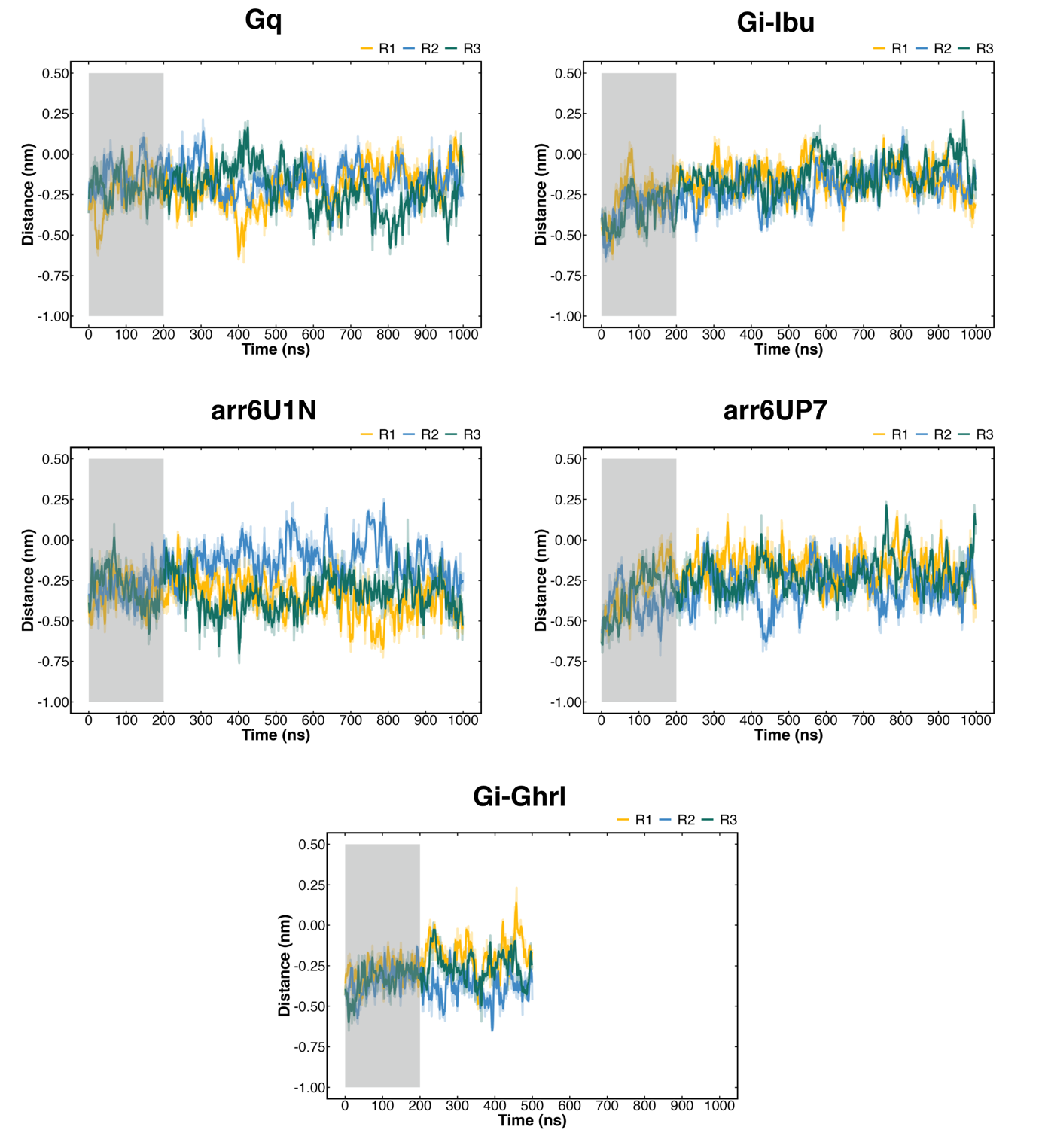
**

**Figure S2**: ***Z*-component of the distance between the GHSR centre (defined as the centre of mass of Asp^2.50^) and the average position of the membrane P atoms throughout the MD simulation.** Replicas are colour-coded as follows: R1, yellow; R2, blue; and R3, green. The gray area marks the first 200 ns and is excluded from the remaining analysis.

**
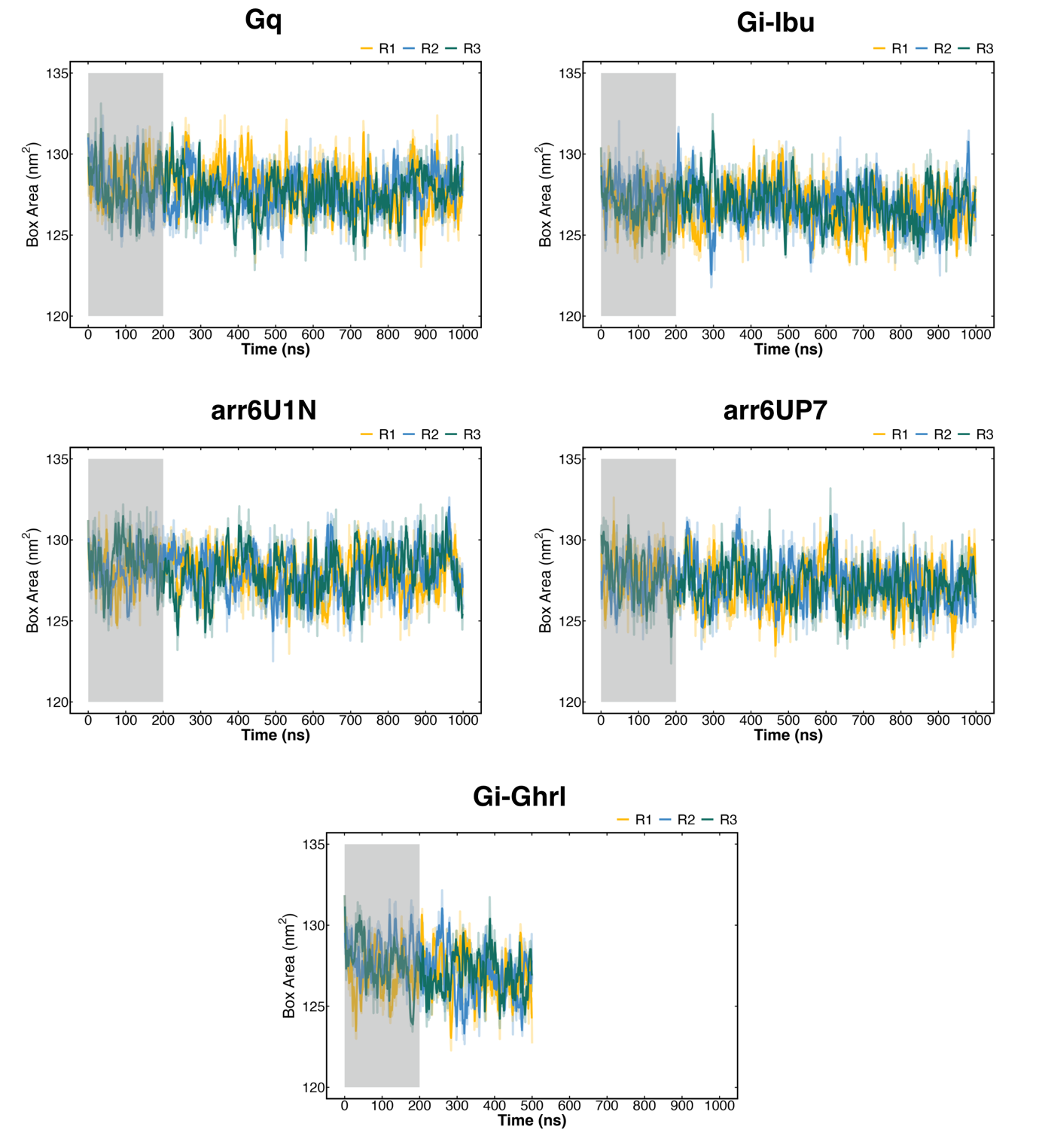
**

**Figure S3:** **Area of system box (*XY* plane) throughout the MD simulations.** Replicas are colour-coded as follows: R1, yellow; R2, blue; and R3, green. The gray area marks the first 200 ns and is excluded from the remaining analysis.


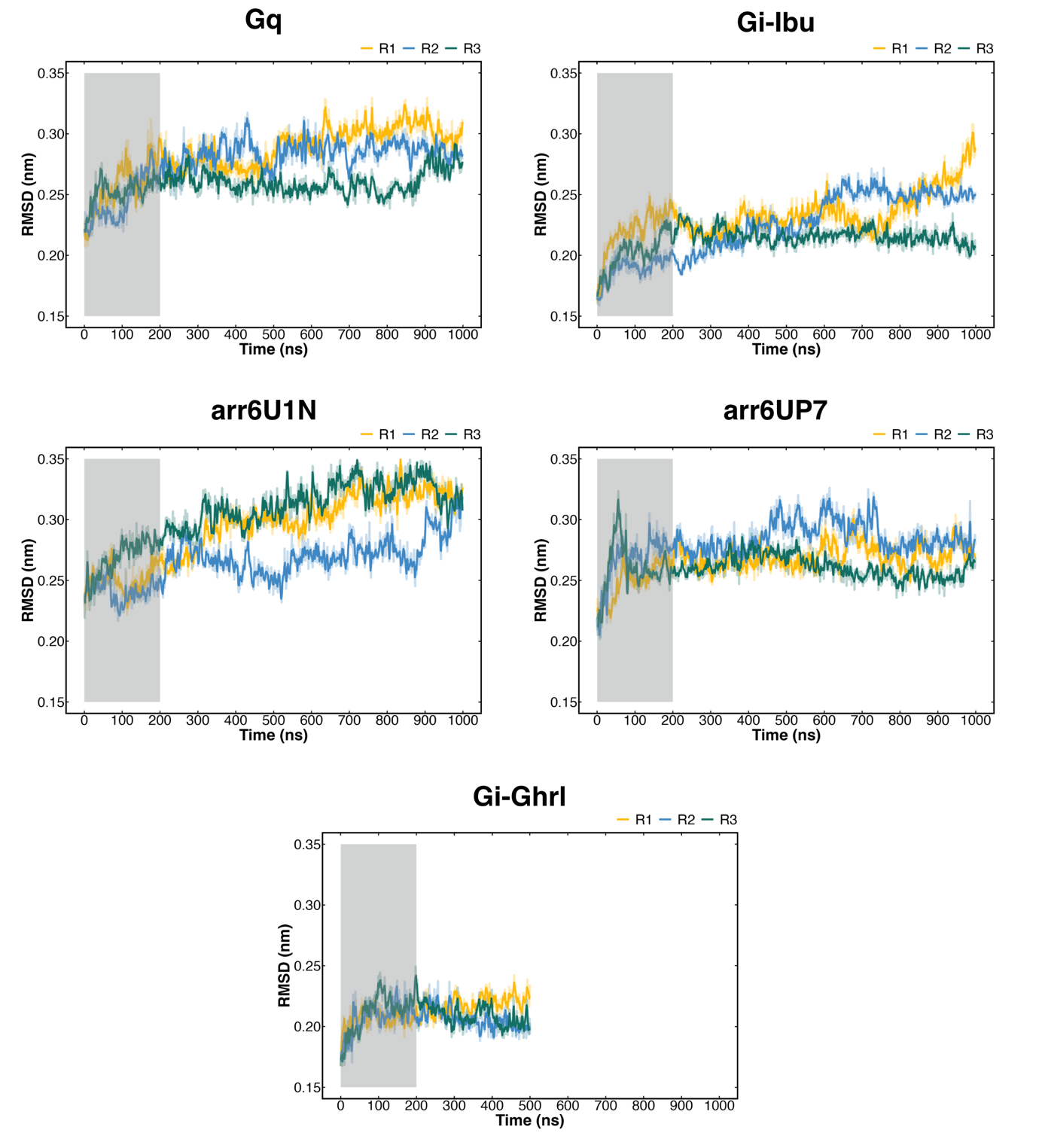


**Figure S4:** **RMSD of TM region of GHSR for five simulated systems.** throughout the MD simulation. Replicas are colour-coded as follows: R1, yellow; R2, blue; and R3, green. The gray area marks the first 200 ns and is excluded from the remaining analysis.


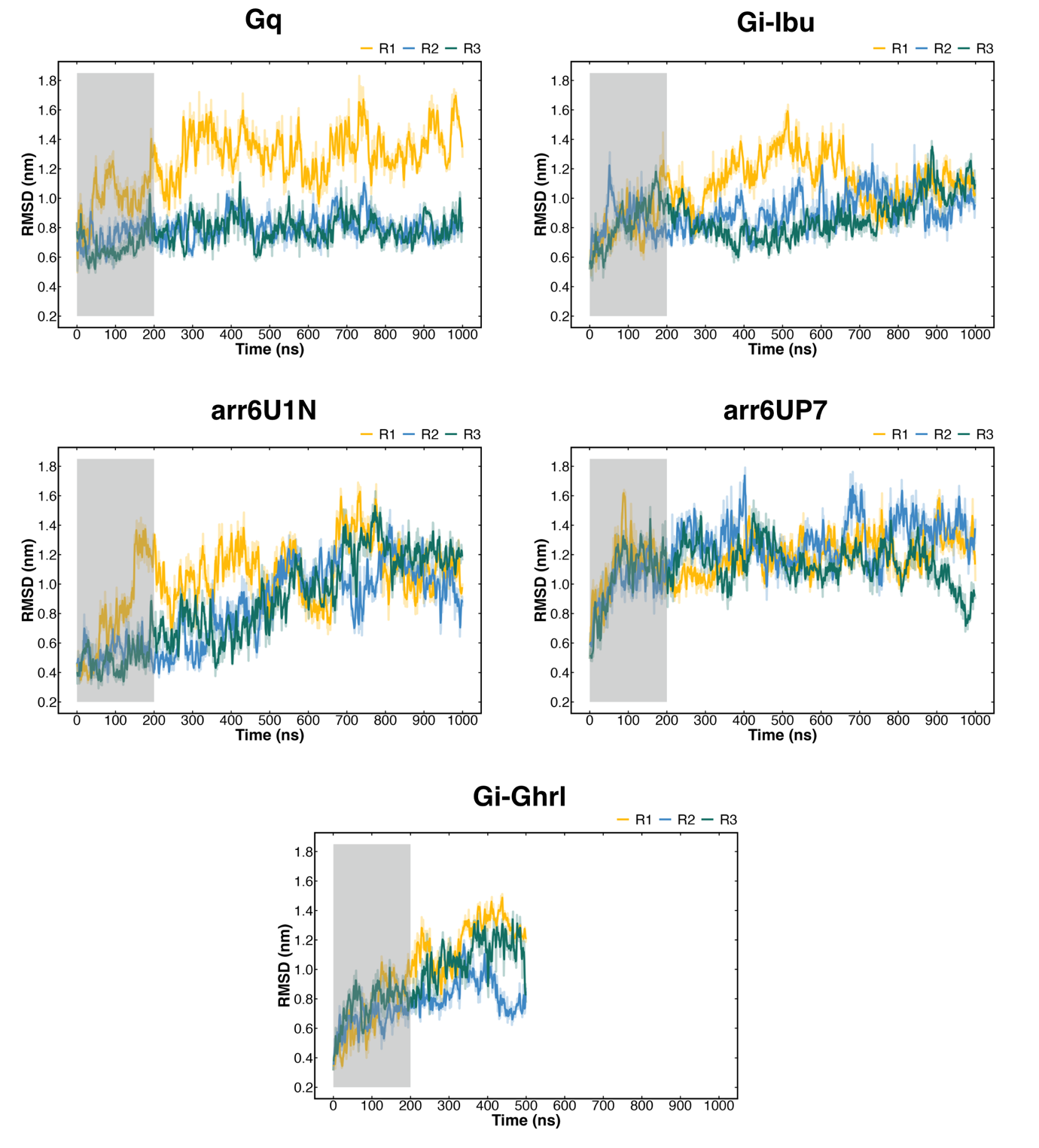


**Figure S5:** **RMSD of partner proteins in five different simulated systems throughout the MD simulations.** Replicas are colour-coded as follows: R1, yellow; R2, blue; and R3, green. The gray area marks the first 200 ns and is excluded from the remaining analysis.

**
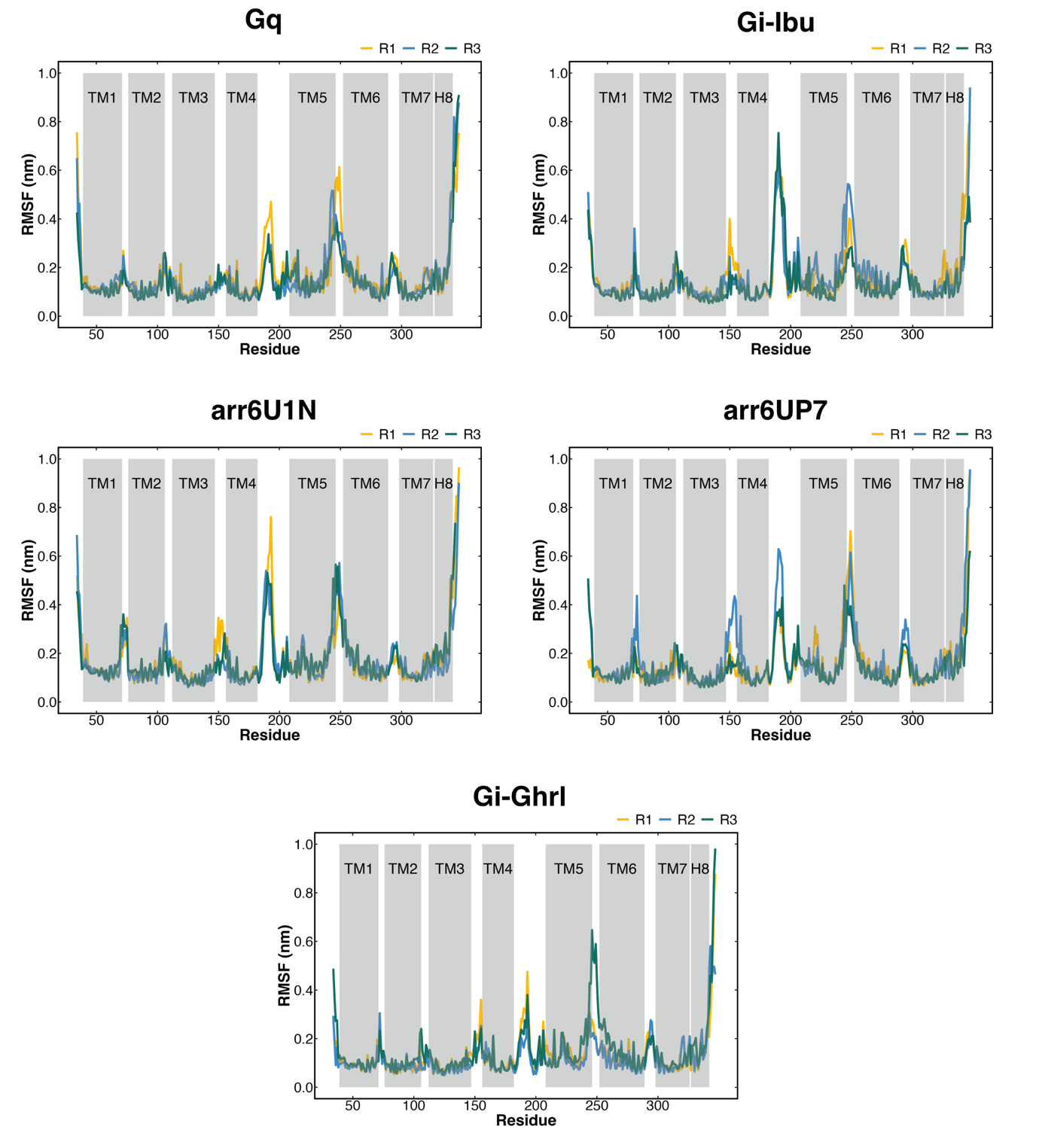
**

**Figure S6:** **RMSF of GHSR for the five simulated systems.** The different TM regions and H8 are highlighted in grey. Replicas are colour-coded as follows: R1, yellow; R2, blue; and R3, green.

**
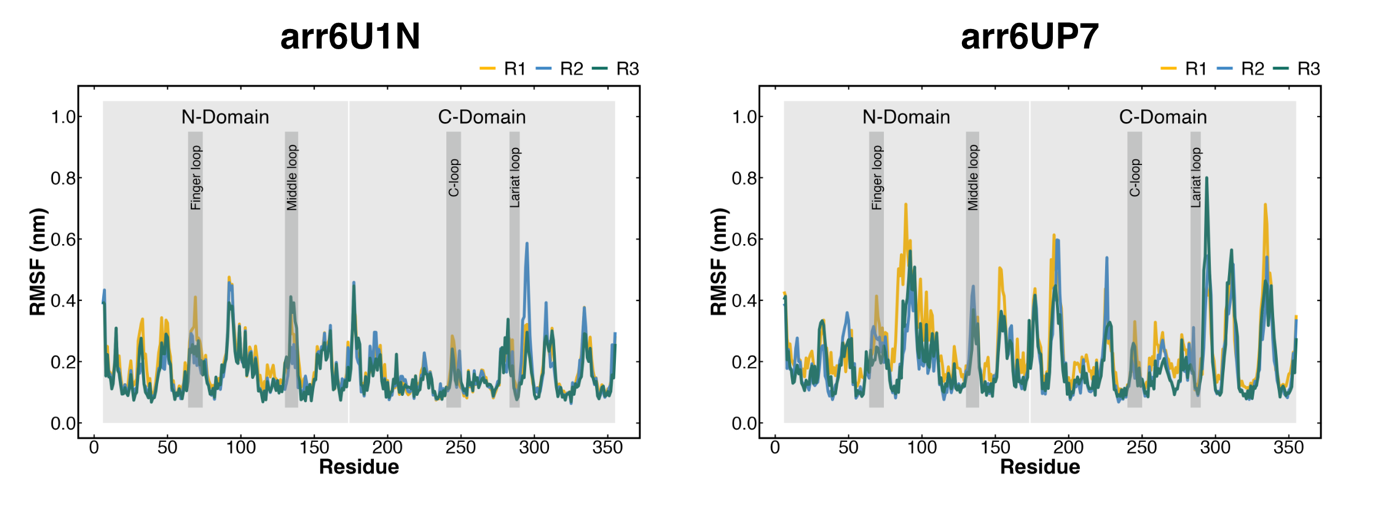
**
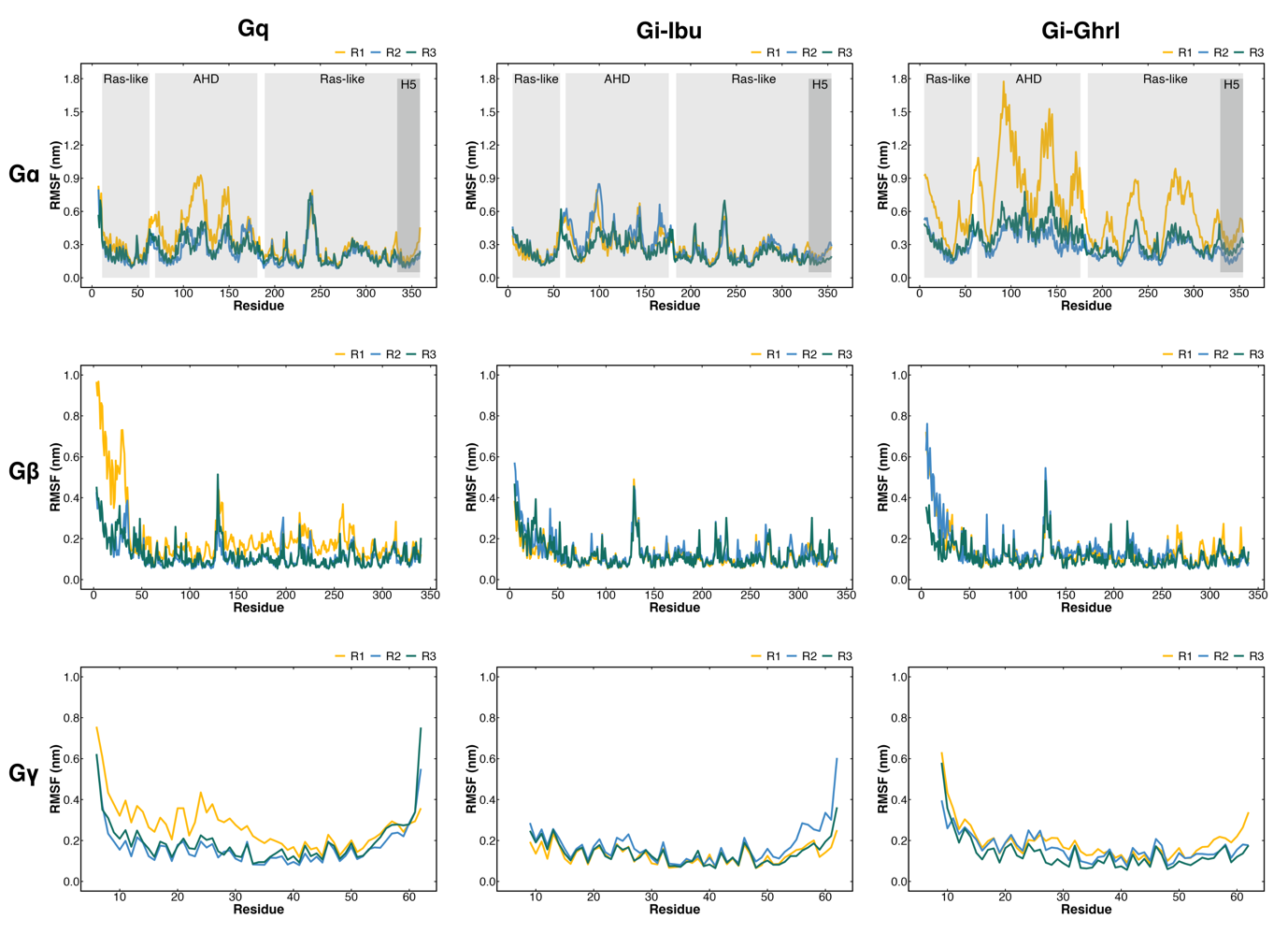
 **Figure S7:** **RMSF of G protein for three different simulated systems.** Replicas are colour-coded as follows: R1, yellow; R2, blue; and R3, green.

**Figure S8:** **RMSF of arrestin for the two different simulated systems.** Replicas are colour-coded as follows: R1, yellow; R2, blue; and R3, green.


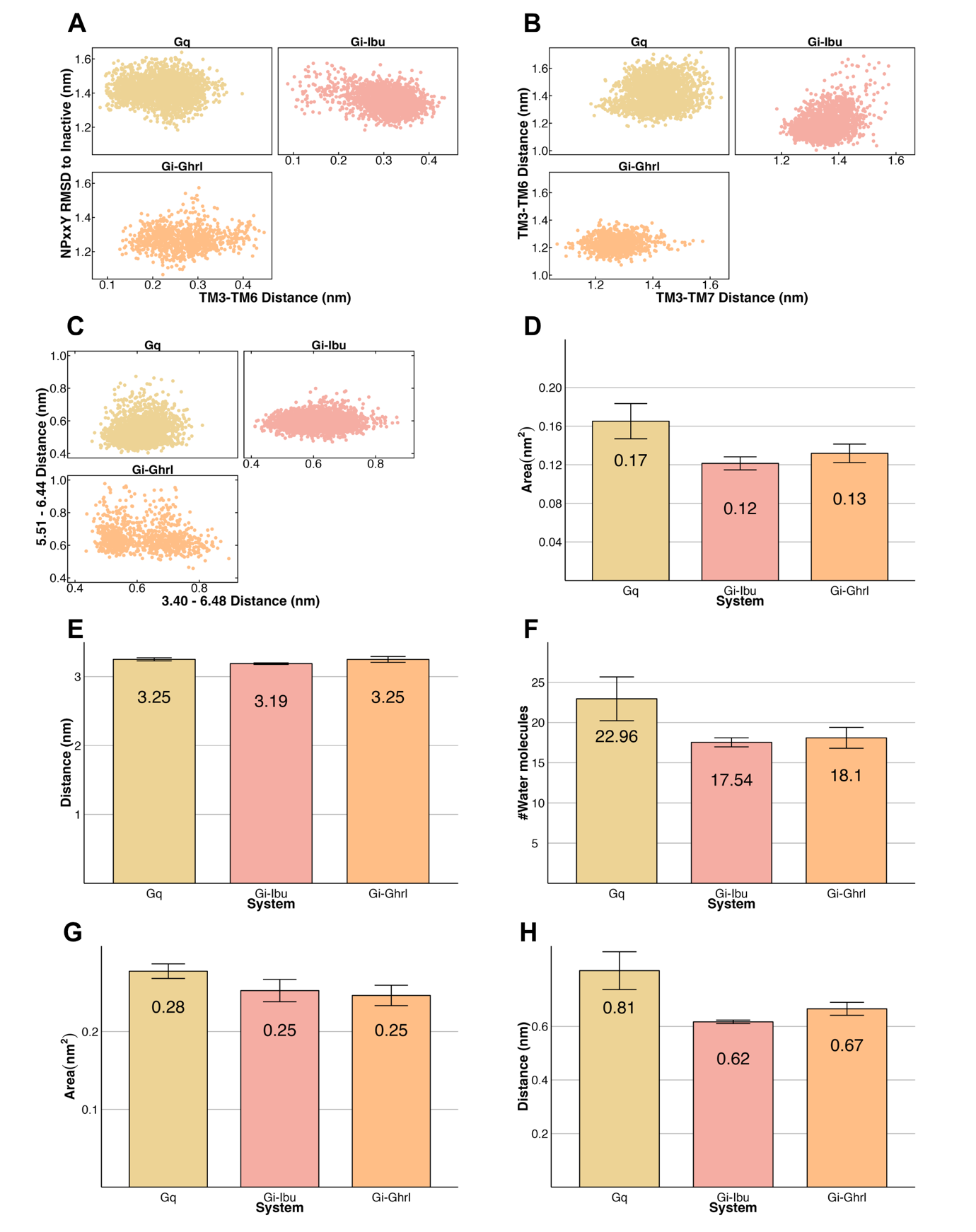


**Figure S9: Analysis of GPCR activation sites in G protein-bound systems**. A - Comparison of the TM3-TM6 distance (measured between Cα of Arg141^3.50^ and Val262^6.34^) and the RMSD of NPxxY motif’s backbone to the inactive structure; B - Comparison of the TM3-TM6 distance (measured between Cα of Arg141^3.50^ and Val262^6.34^) and the TM3-TM7 distance (measured between Cα of Arg141^3.50^ and Tyr323^7.53^); C - Comparison of distances within Transmission Switch, represented as the distances between Val131^3.40^ and Trp276^6.48^ side chains and Val225^5.51^ and Phe272^6.44^ side chains; D - Na^+^ pocket area, calculated using the triangle of distances between Asp89^2.50^, Thr130^3.39^ and Asn319^7.49^; E - Distance between Asp89^2.50^ and the closest Na^+^ ion; F - Number of water molecules within 8 Å of residue Asp89^2.50^; G - Hydrophobic Lock area, calculated using the triangle of distances between Ile134^3.43^, Val268^6.40^, and Val269^6.41^; H - Distance between side chains of Ile134^3.43^ and Tyr323^7.53^. Bars represent the mean ± SEM of each system. The systems were colour-coded as G_q_, yellow; G_i_-Ibu, red, and G_i_-Ghrl, orange.

**
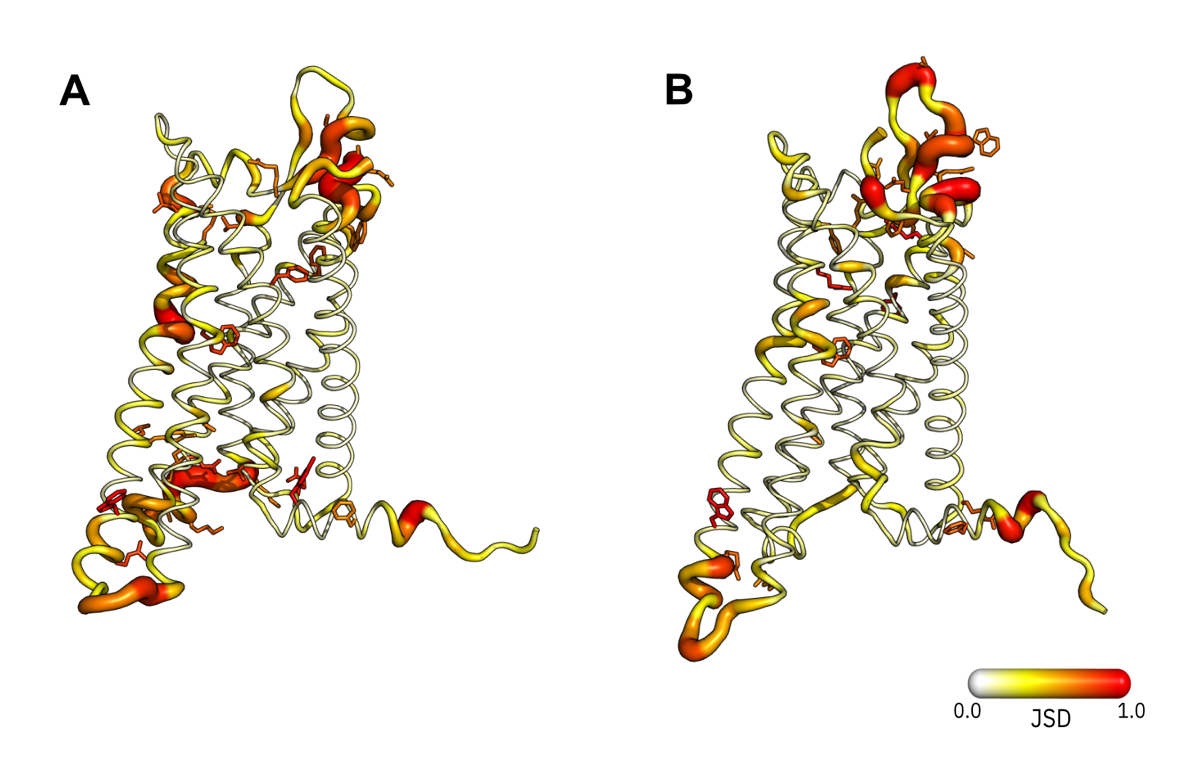
**

**Figure S10: Comparison of torsion angles of the backbone and side chains via JSD between the G protein systems.** A. Comparison of G_q_-bound and G_i_-Ghrl systems. B - Comparison between the two G_i_-bound systems (G_i_-Ibu and G_i_-Ghrl). The backbone and side chains are coloured according to the JSD values from white (0.0) to red (1.0). Side chains are shown for residues with a JSD higher than 0.6. The structures were visualised using PyMol software.

**
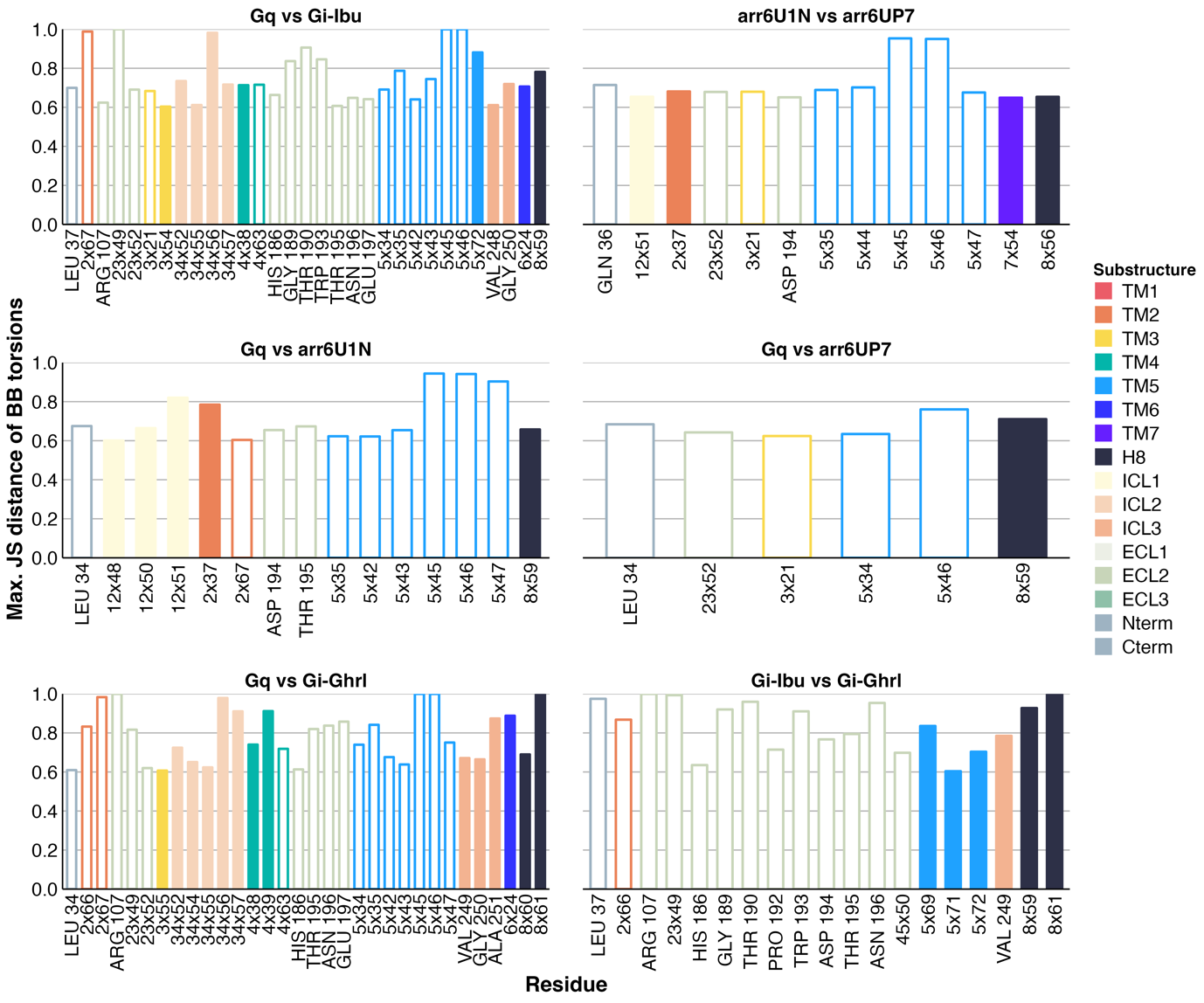
**

**Figure S11:** **JSD values for backbone torsion per GHSR residue.** Only residues with JSD > 0.6 are shown. The bars are coloured according to the substructure. The filled bars correspond to intracellular residues, whereas the non-filled bars correspond to extracellular residues.


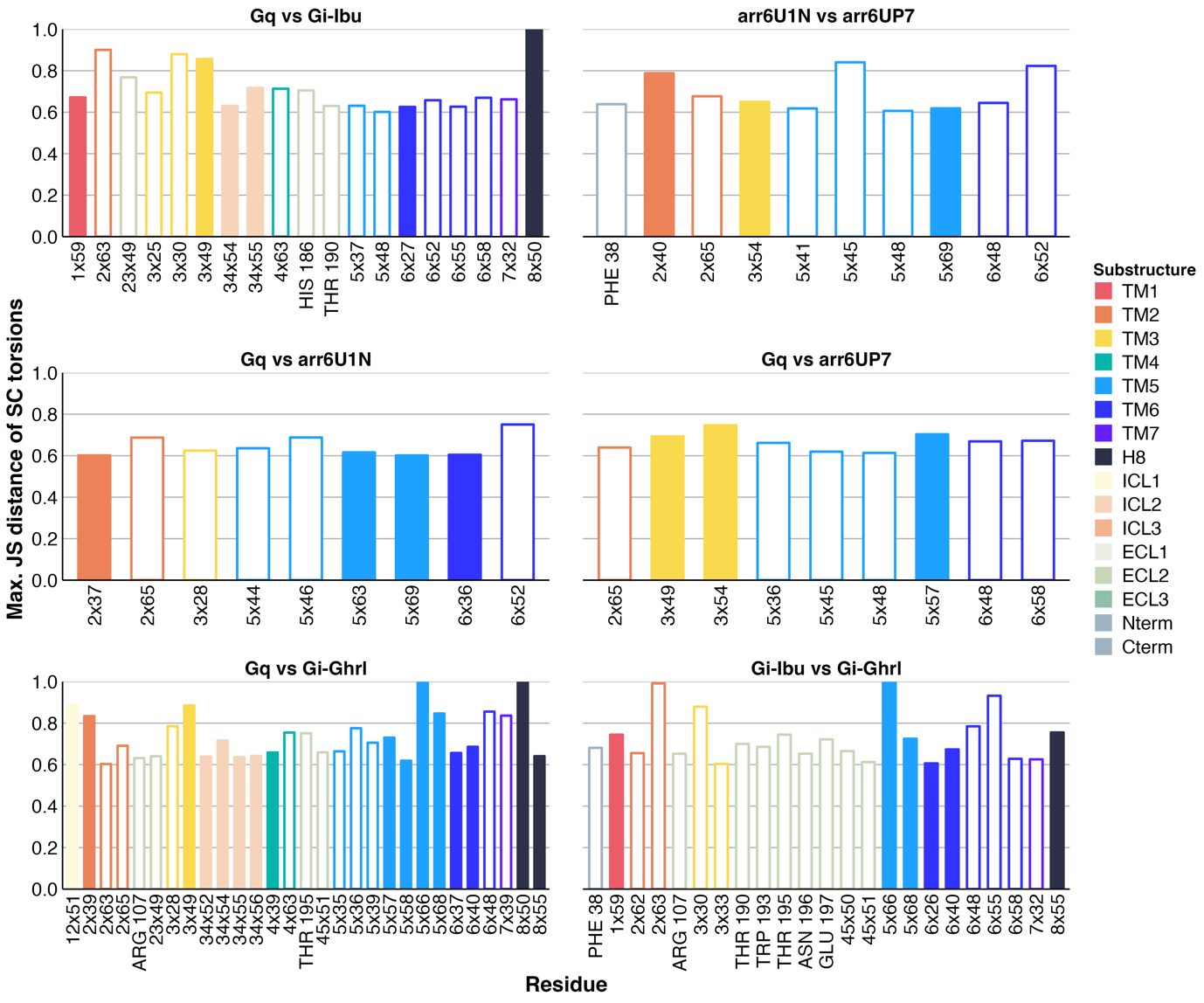


**Figure S12:** **JSD values for side-chain torsion per GHSR residue.** Only residues with JSD > 0.6 are shown. Bars are coloured according to the substructure. The filled bars correspond to intracellular residues, whereas non-filled bars correspond to extracellular residues.


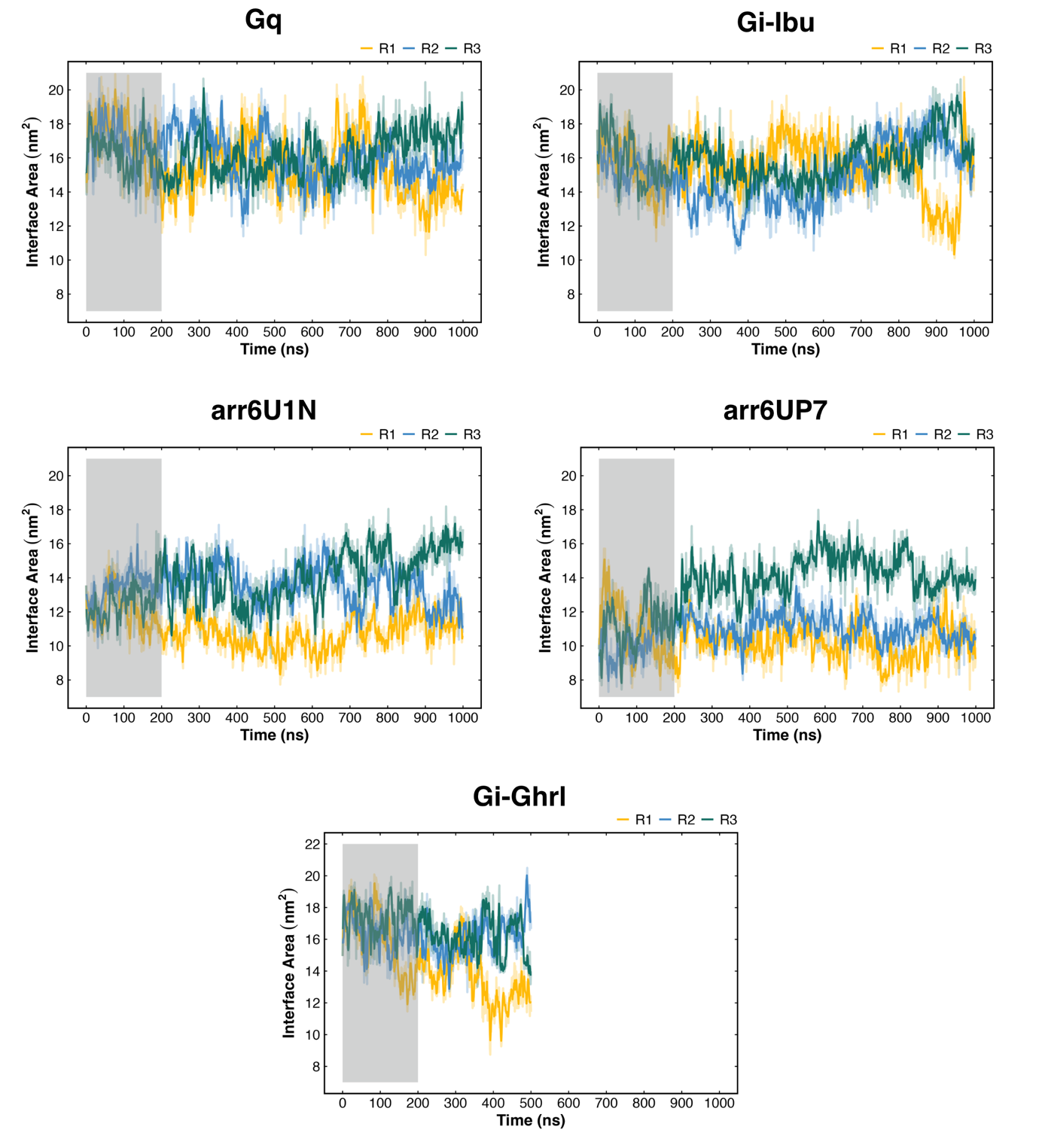


**Figure S13: Interface area of GHSR for the five different complexes over simulation time.** The interface area was calculated using the SASA of the receptor in the presence and absence of the partner (see Methods section for further details). Replicas are colour-coded as follows: R1, yellow; R2, blue; and R3, green. The gray area marks the first 200 ns, which were excluded from the rest of the analysis.

**
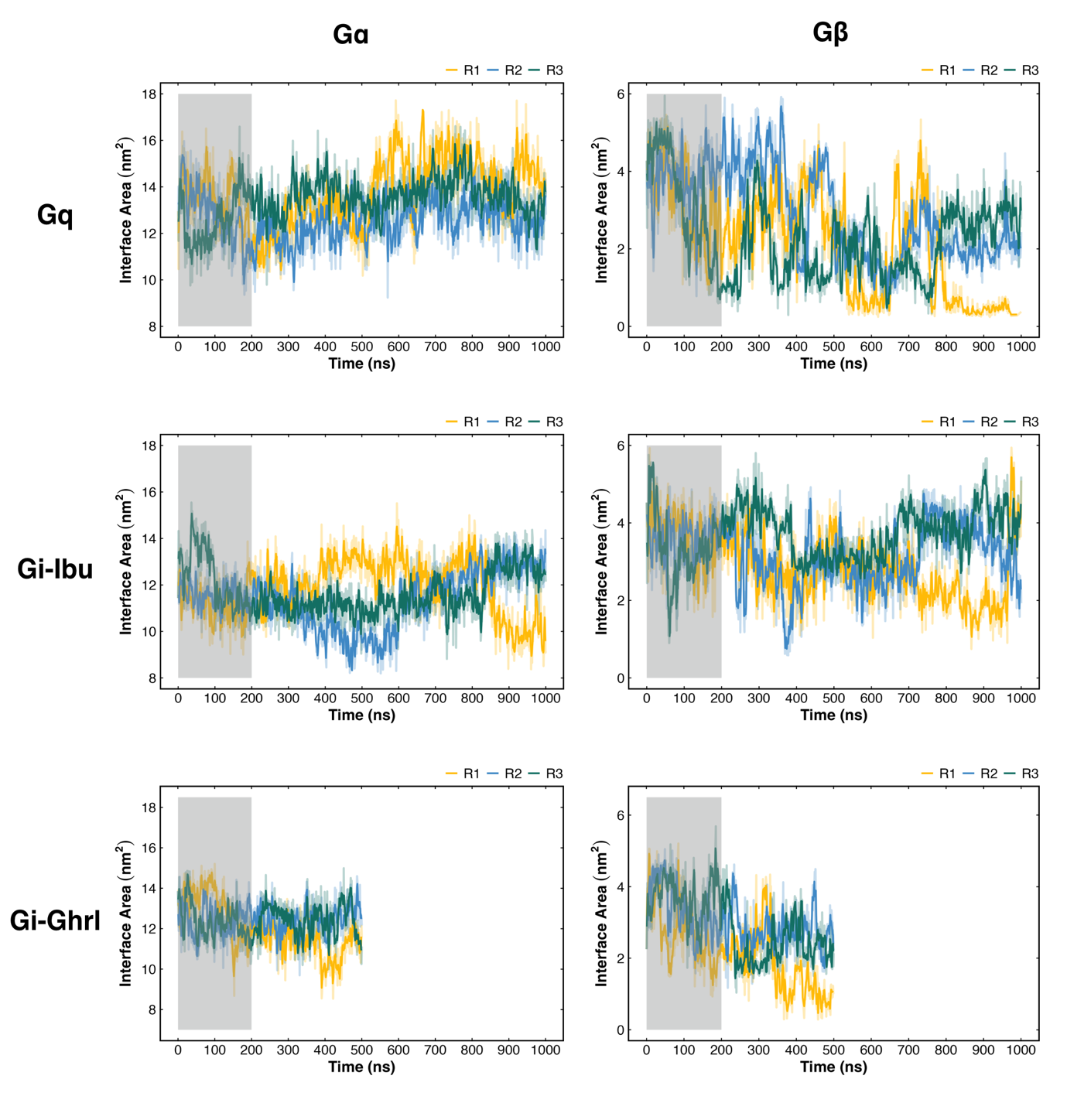
Figure S14:** **Interface area of the G protein on three different complexes over simulation time.** The interface area was calculated using the SASA of the G protein in the presence and absence of GHSR (see Methods for more details). Replicas are colour-coded as follows: R1, yellow; R2, blue; and R3, green. The gray area marks the first 200 ns, which were excluded from the rest of the analysis.


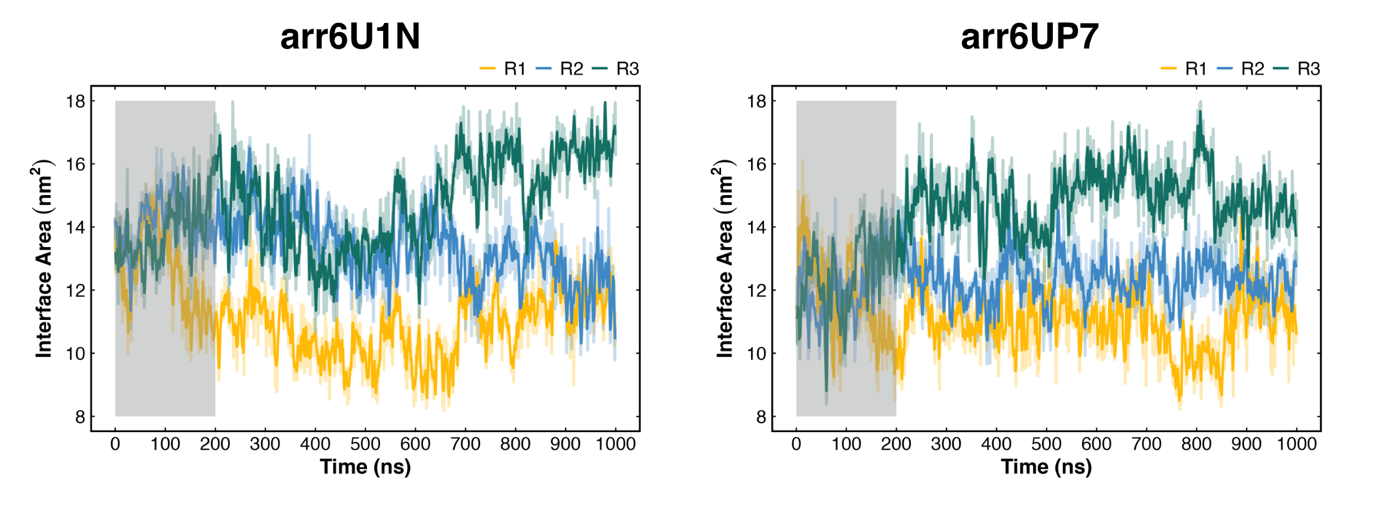


**Figure S15:** **Interface area of arrestin for two different complexes over simulation time.** The interface area was calculated using the SASA of arrestin in the presence and absence of GHSR (see Methods section for more details). Replicas are colour-coded as follows: R1, yellow; R2, blue; and R3, green. The gray area marks the first 200 ns, which were excluded from the rest of the analysis.

**
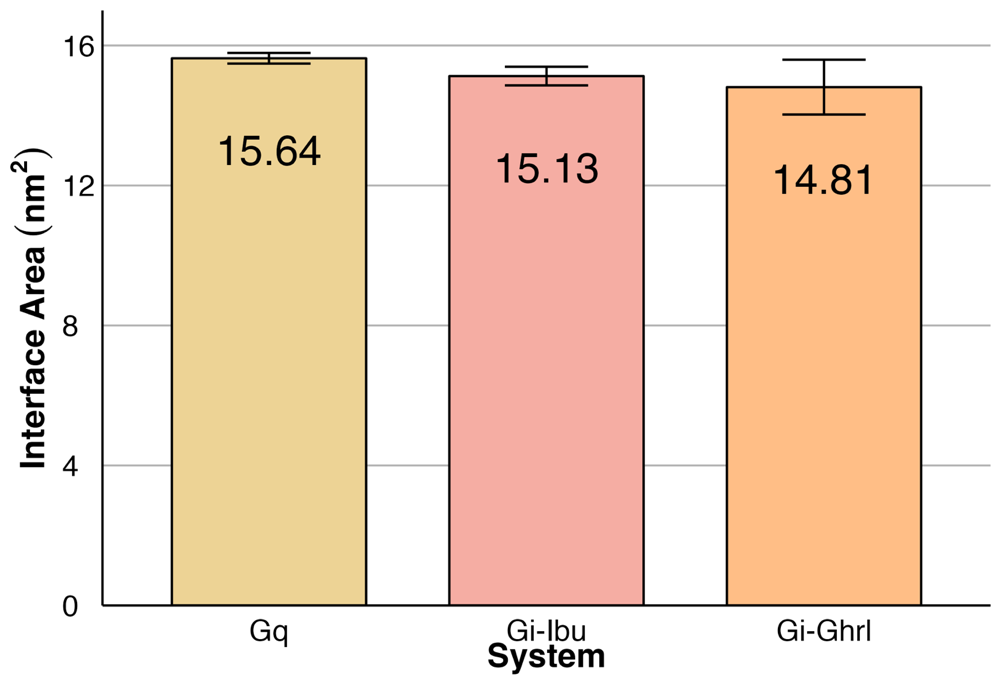
**

**Figure S16:** **Interface area of GHSR – G protein systems.** Replicates are summarised as mean ± standard error of the mean. Systems are colour-coded: G_q_, yellow; G_i_-Ibu, red; and G_i_-Ghrl, orange.


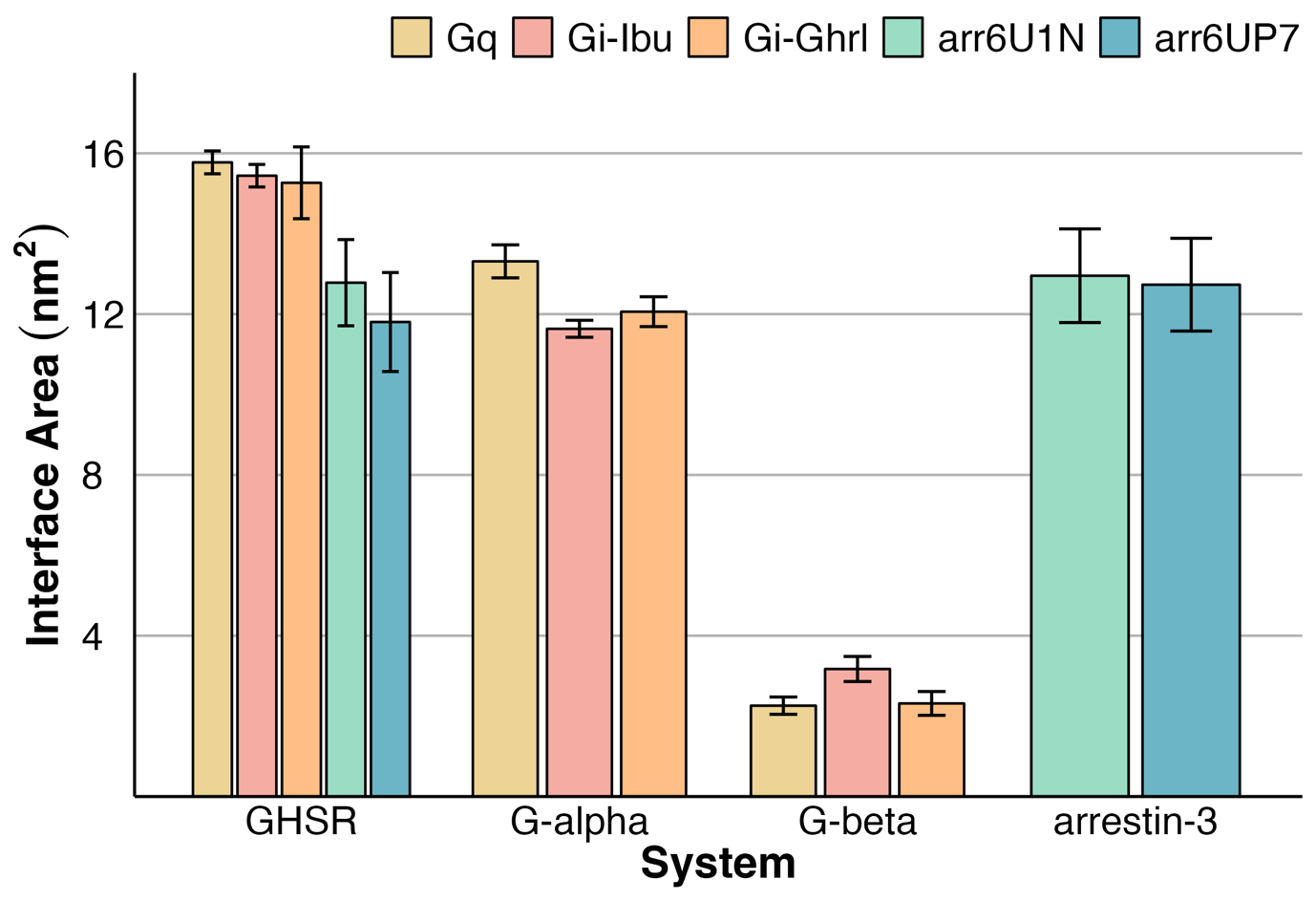


**Figure S17**: **Interface area per protein for each GHSR partner system.** Replicates are summarised as the mean ± standard error of the mean. Systems are colour-coded as follows: G_q_, yellow; G_i_-Ibu, red; G_i_-Ghrl, orange; arr6U1N, green; and arr6UP7, blue.


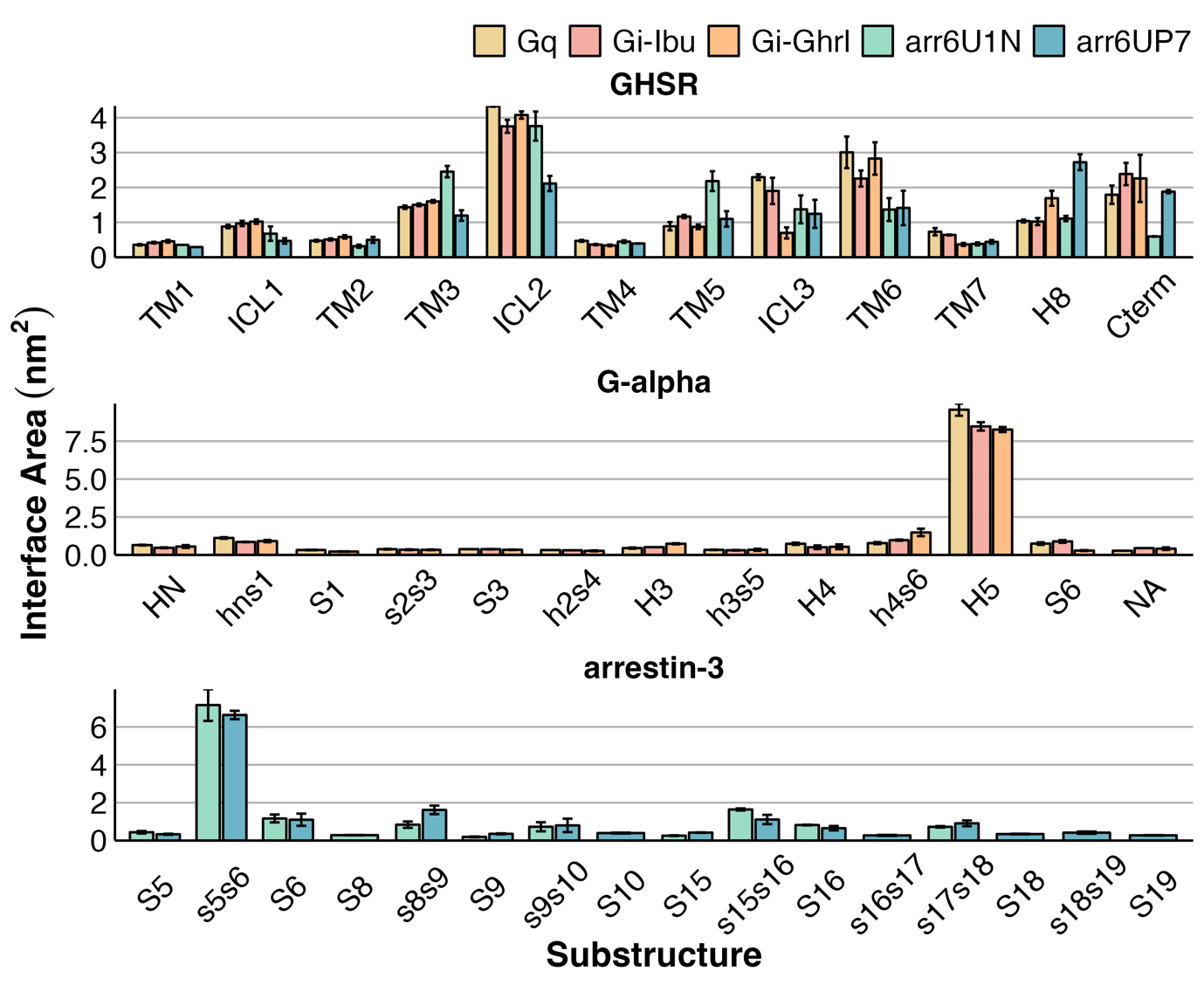


**Figure S18: Interface area divided by the substructure of each GHSR - partner system.** Replicates are summarised as the mean ± standard error of the mean. Systems are colour-coded as follows: G_q_, yellow; G_i_-Ibu, red; G_i_-Ghrl, orange; arr6U1N, green; and arr6UP7, blue.


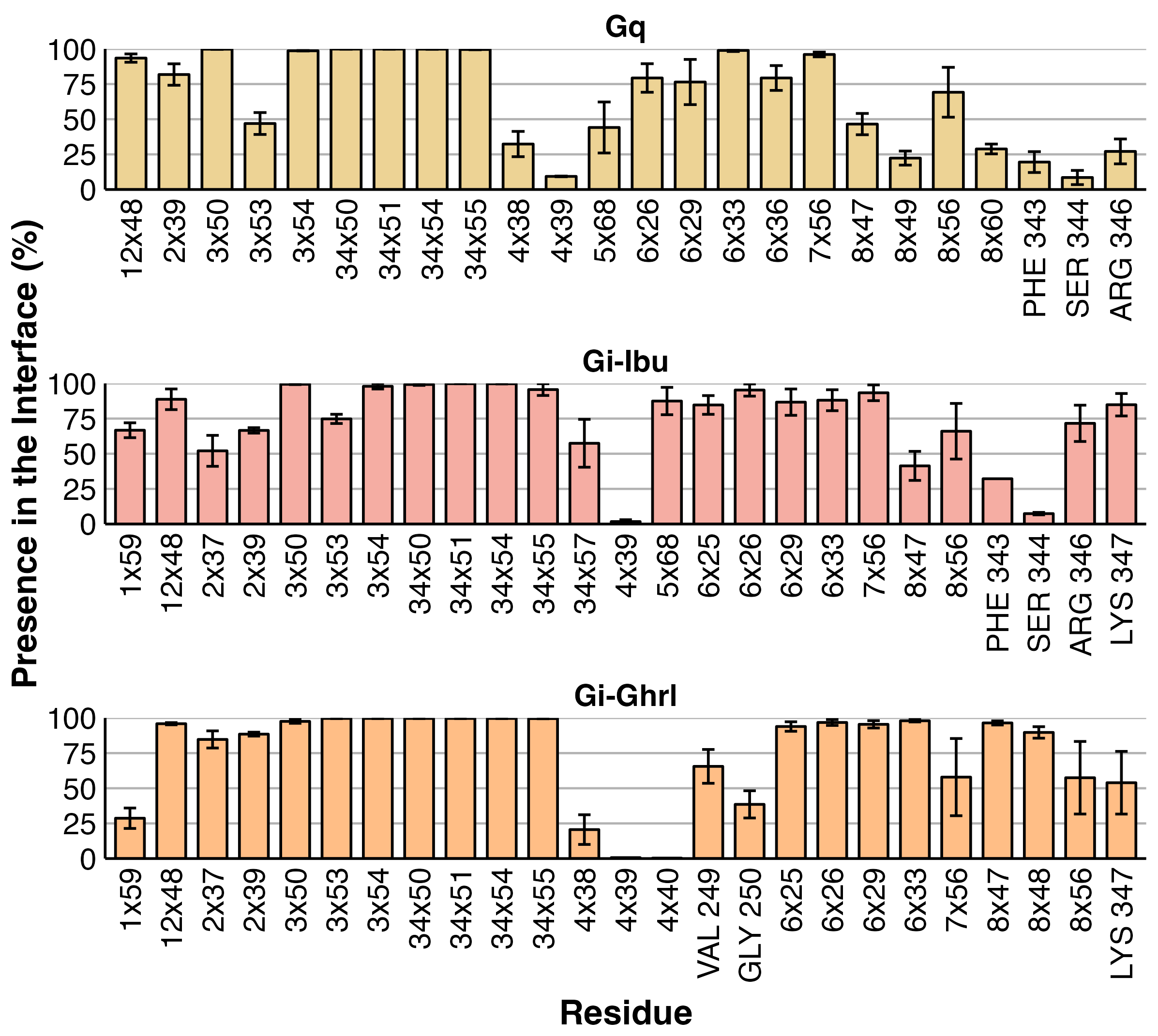


**Figure S19:** **Original decoy interfacial residues for GHSR in G protein systems.** Replicates are summarised as mean ± standard error of the mean. Systems are colour-coded: G_q_, yellow; G_i_-Ibu, red; and G_i_-Ghrl, orange.


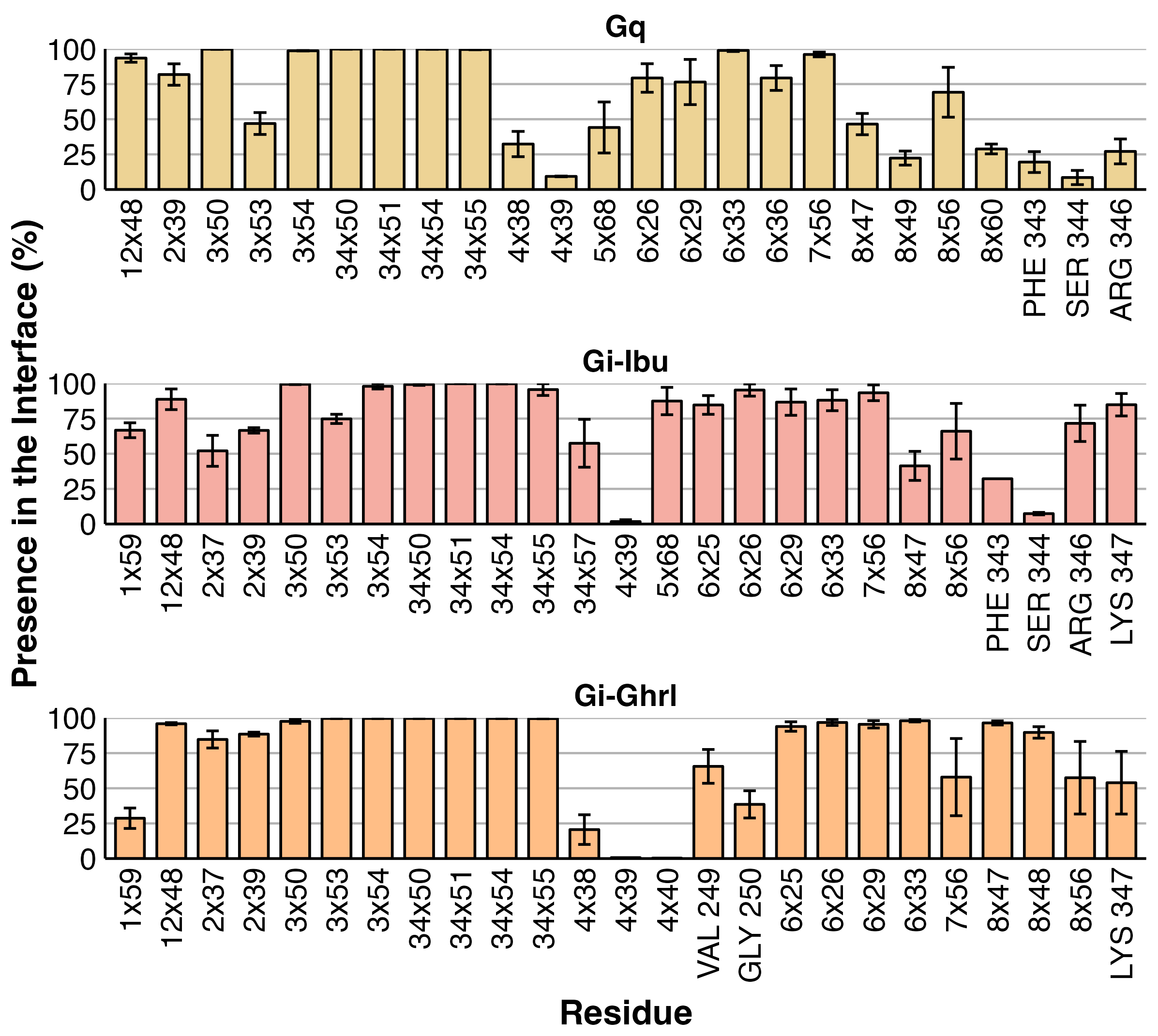


**Figure S20:** **Original decoy interfacial residues of Gα subunit.** Replicates are summarised as the mean ± standard error of the mean. Systems are colour-coded: G_q_, yellow; G_i_-Ibu, red; and G_i_-Ghrl, orange.


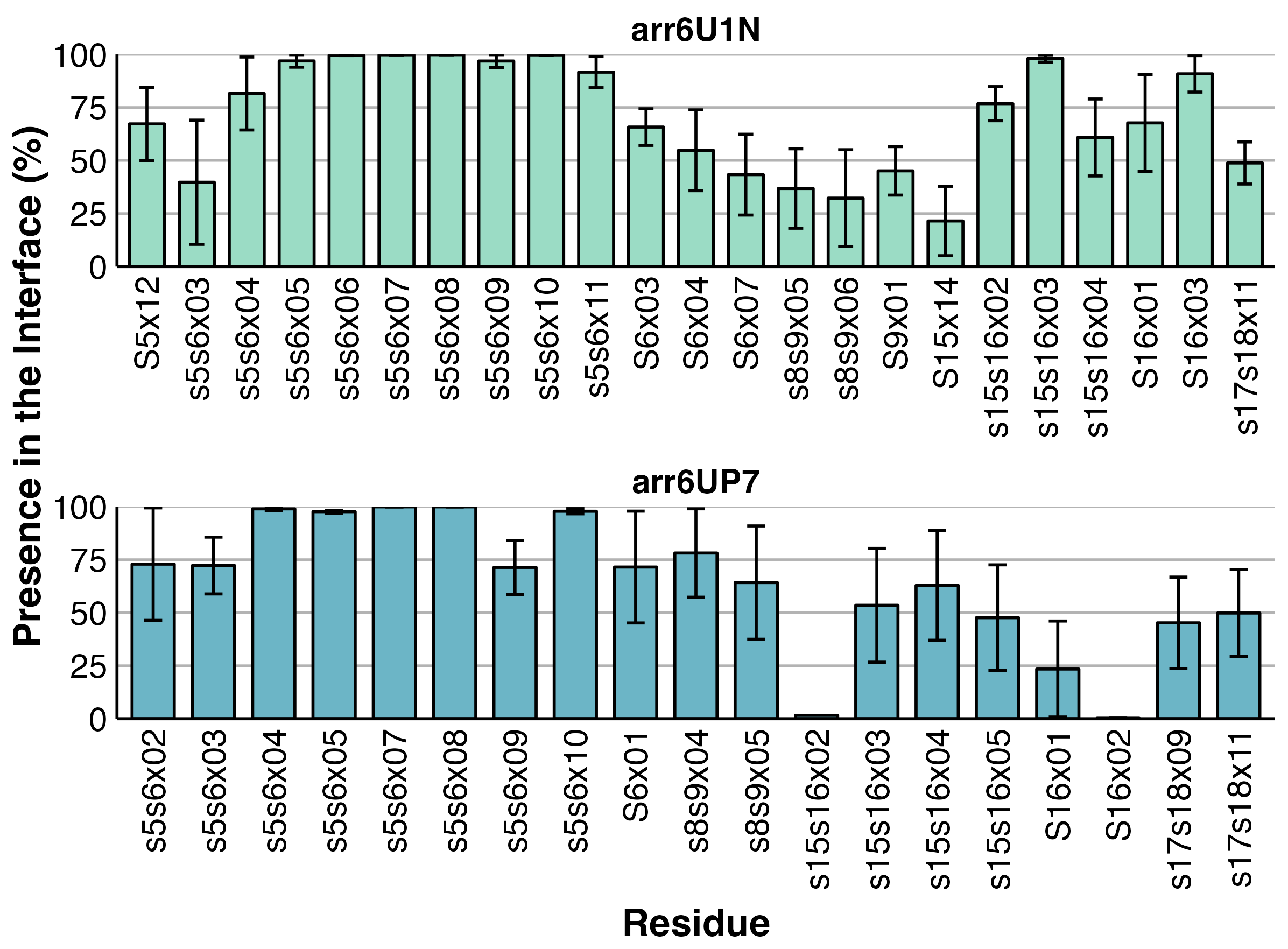


**Figure S21: Original decoy interfacial residues of arrestin proteins.** Replicates are summarised as the mean ± standard error of the mean. Systems are colour-coded: arr6U1N, green; and arr6UP7, blue.


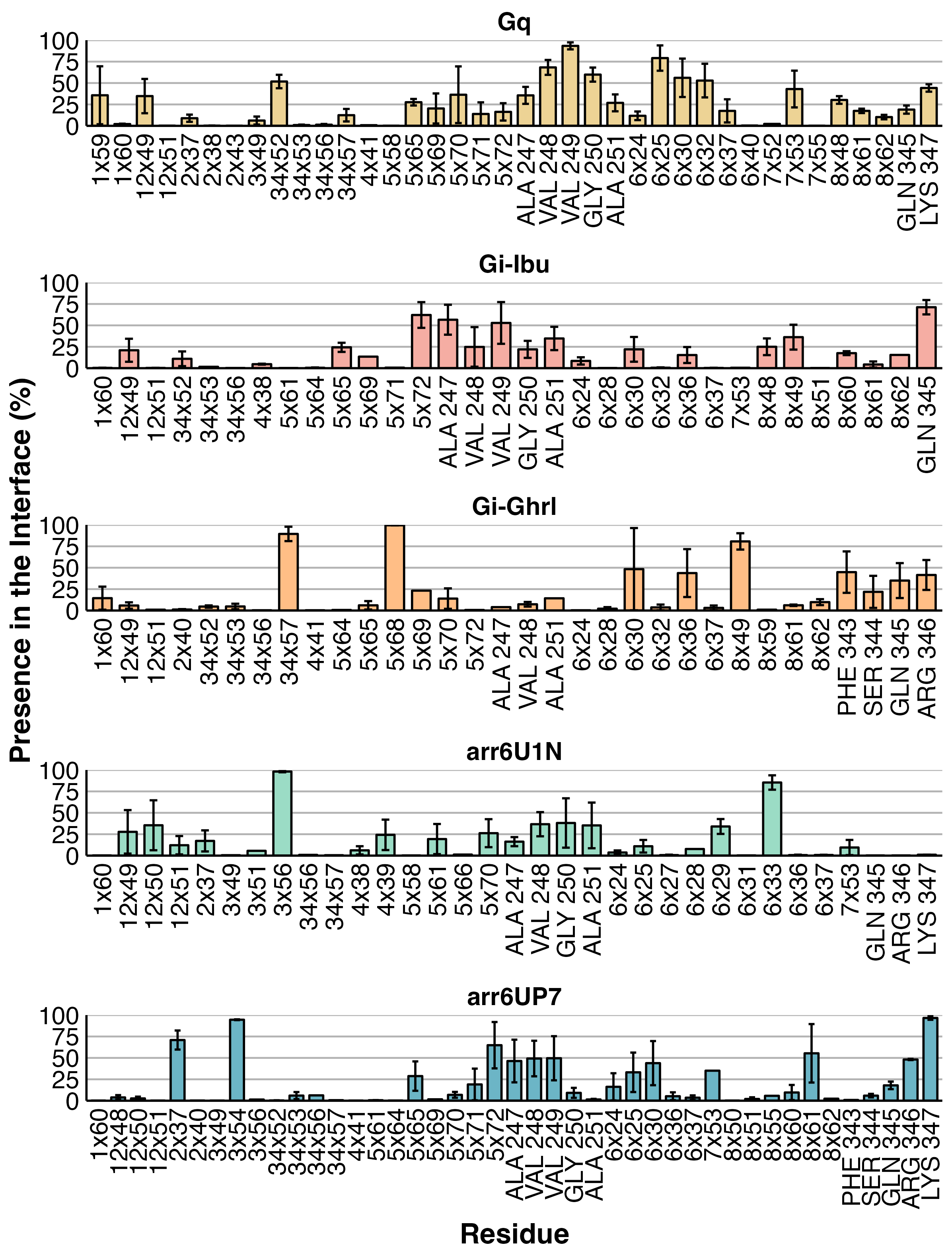


**Figure S22: Additional interfacial residues for GHSR.** Replicates are summarised as the mean ± standard error of the mean. Systems are colour-coded as follows: Systems are colour-coded: G_q_, yellow; G_i_-Ibu, red; and G_i_-Ghrl, orange; arr6U1N, green; and arr6UP7, blue.


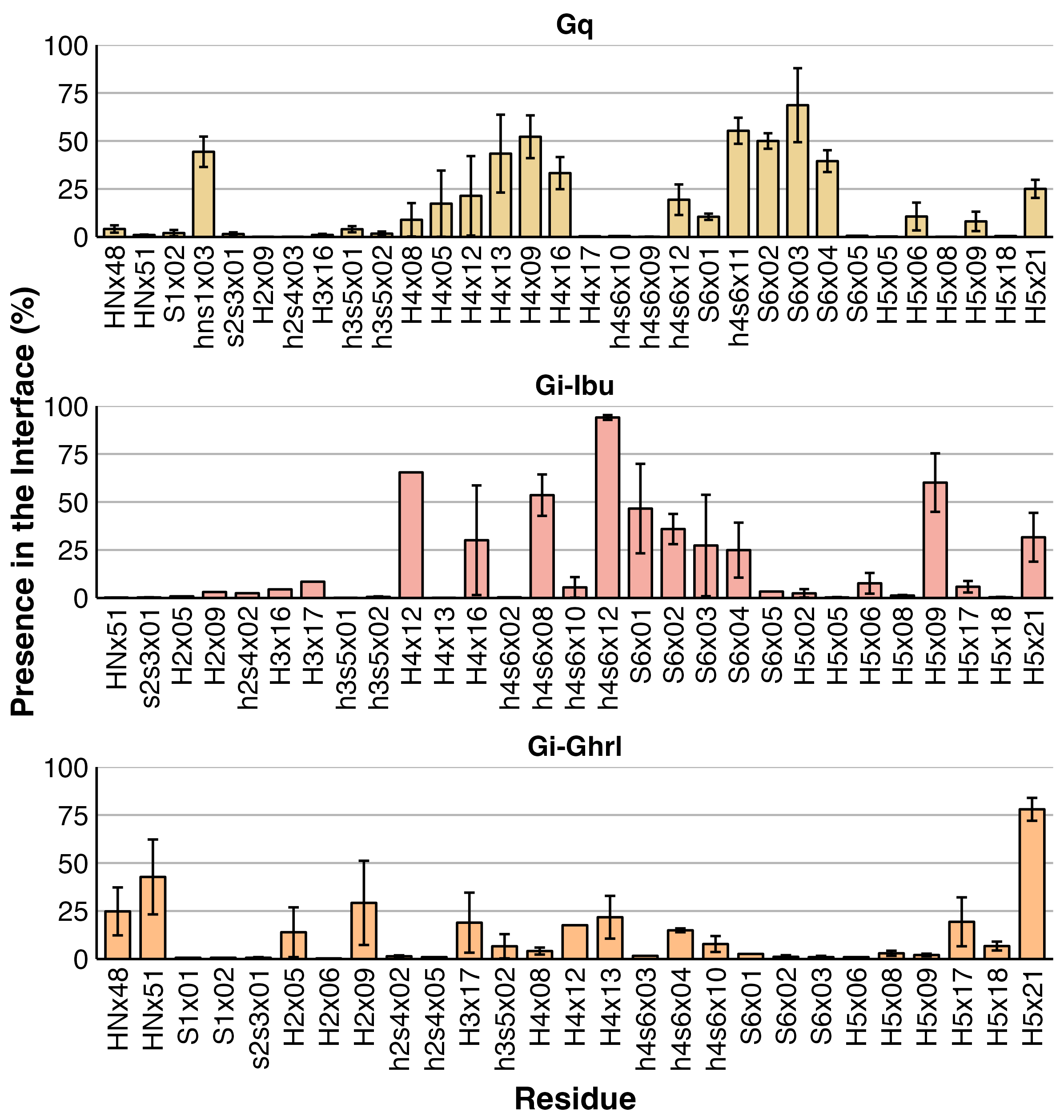


**Figure S23: Additional interfacial residues for Gα subunits.** Replicates are summarised as the mean ± standard error of the mean. Systems are colour-coded: Systems are colour-coded: G_q_, yellow; G_i_-Ibu, red; and G_i_-Ghrl, orange.


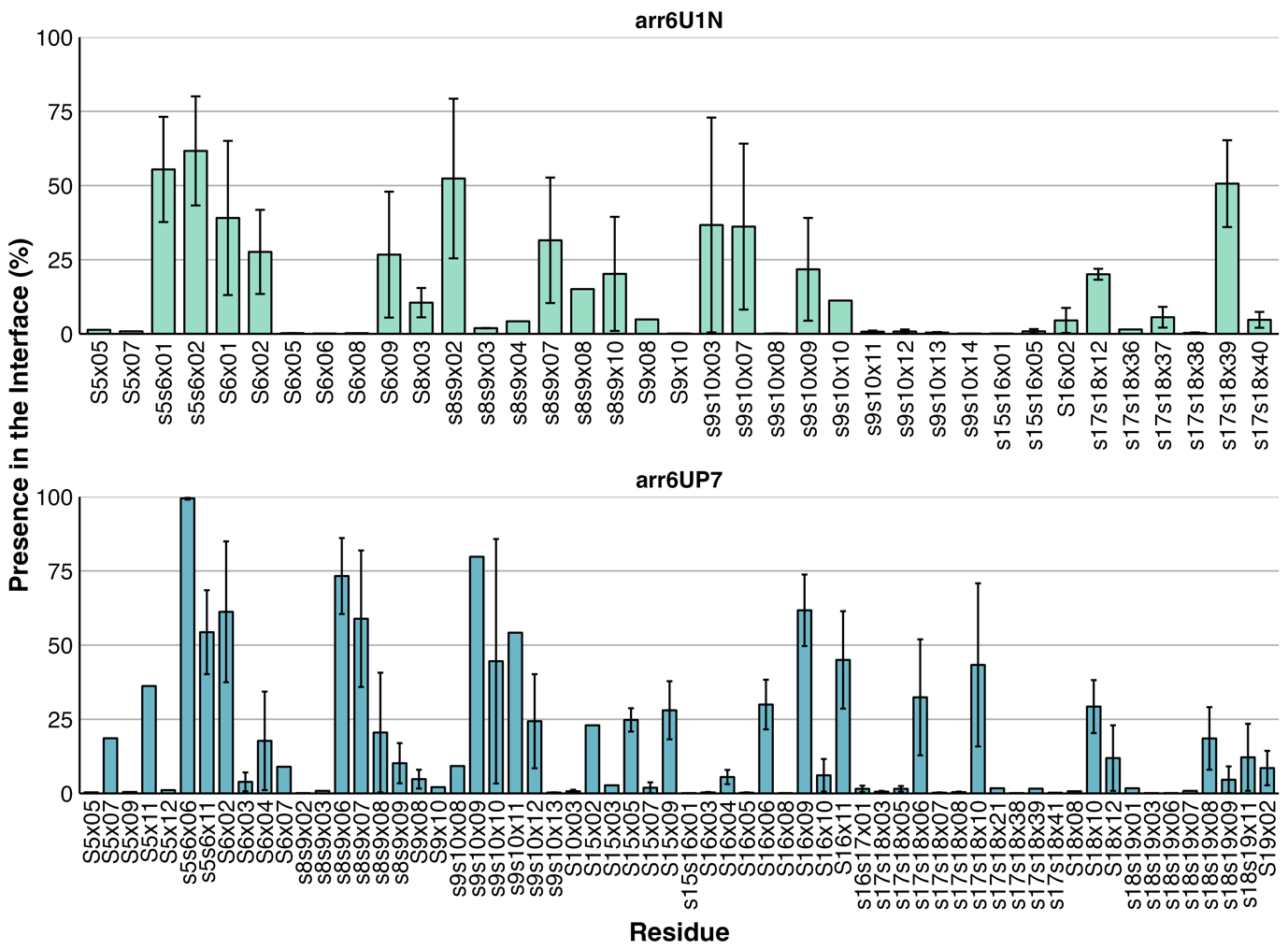


**Figure S24: Additional interfacial arrestin protein residues.** Replicates are summarised as the mean ± standard error of the mean. Systems are colour-coded: arr6U1N, green; and arr6UP7, blue.


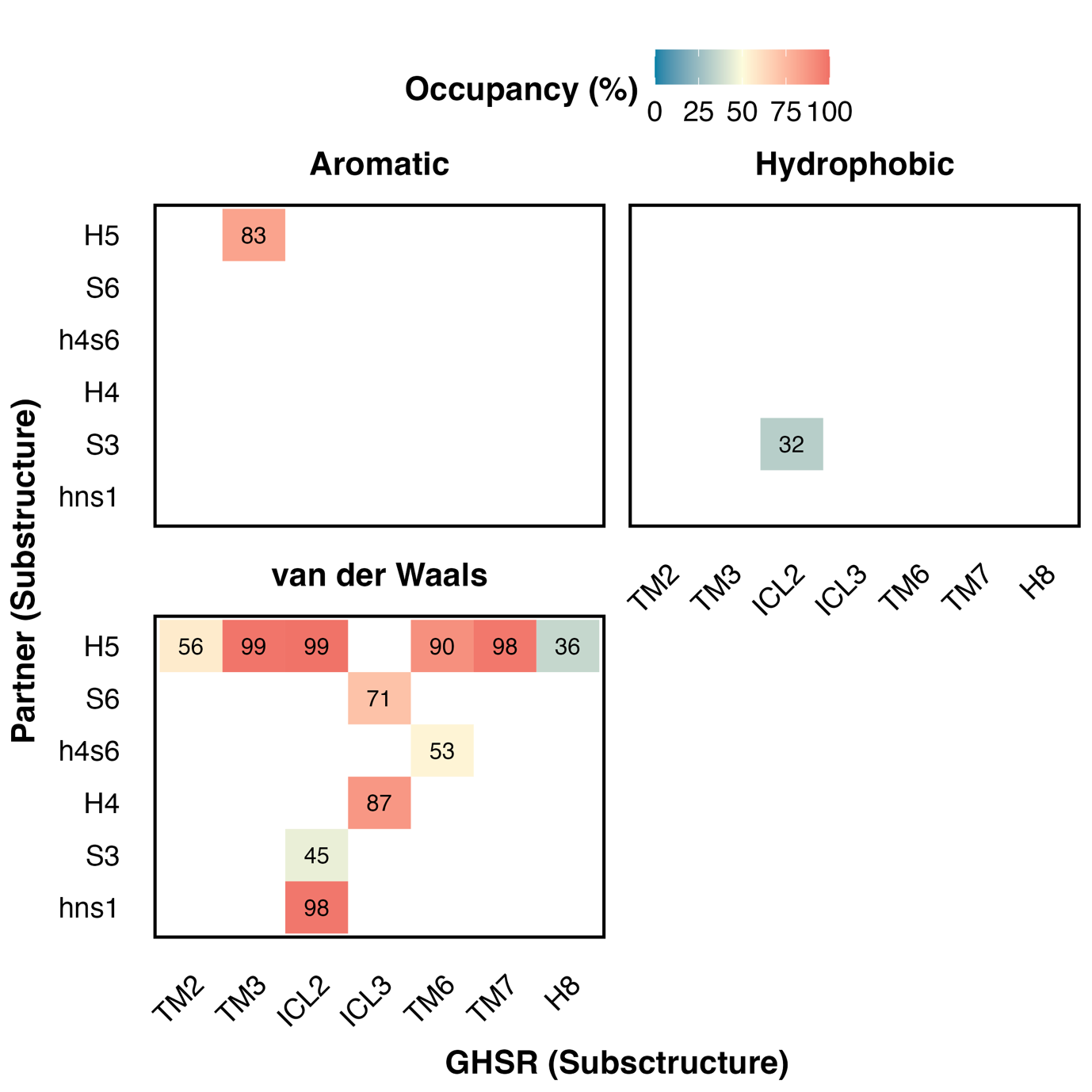


**Figure S25:** **Interaction matrix of the GHSR-G_q_ complex per substructure.** Interactions were divided into different categories (see Methods section for more information). Pairwise interactions established by the residues were determined using the *getcontacts* Python package. The cells were coloured according to the occupancy of the interaction (values inside the cells).


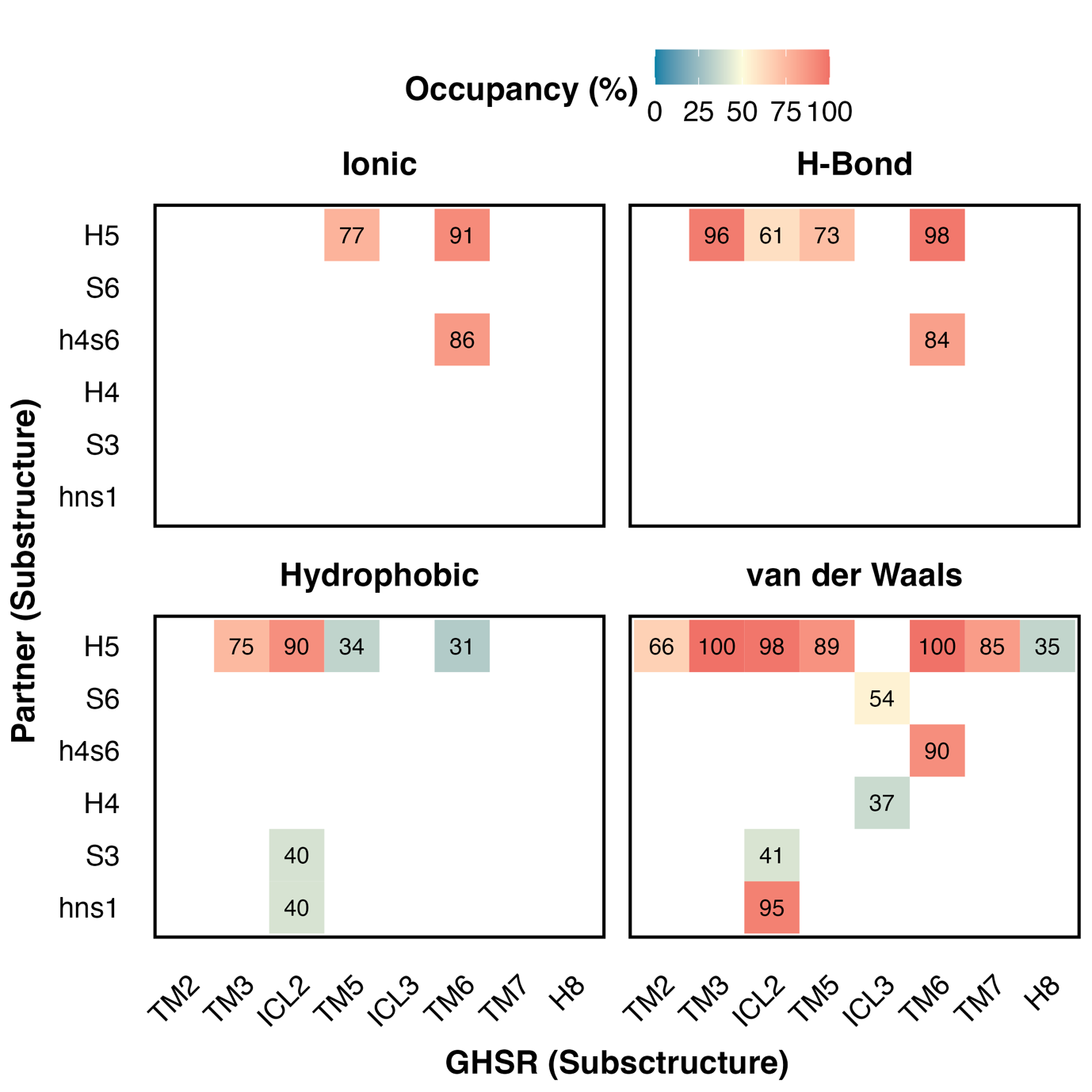


**Figure S26: Interaction matrix of the GHSR-G_i_-Ibu complex per substructure.** Interactions were divided into different categories (see Methods section for more information). Pairwise interactions established by the residues were determined using the *getcontacts* Python package. The cells were coloured according to the occupancy of the interaction (values inside the cells).


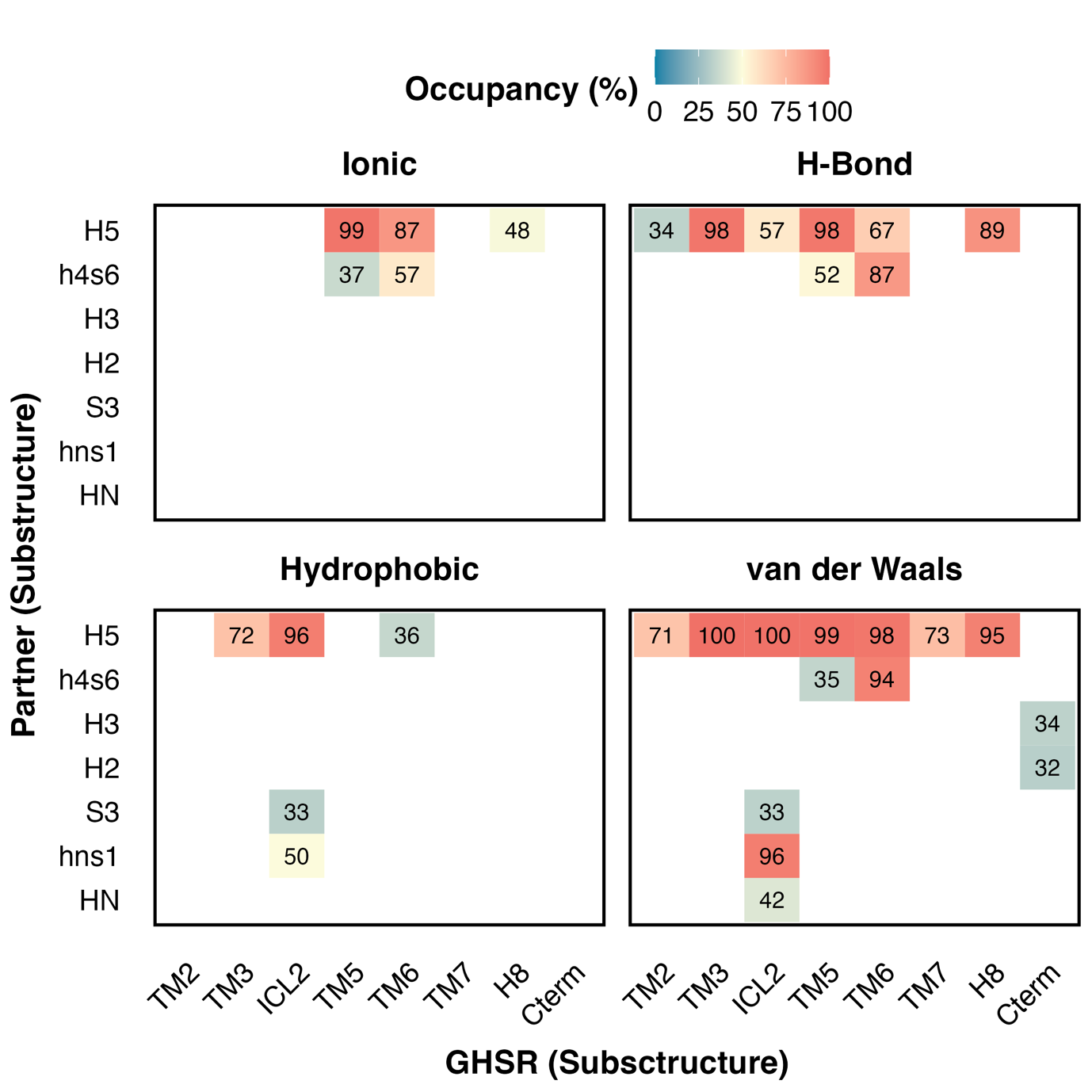


**Figure S27:** **Interaction matrix of the GHSR-G_i_-Ghrl complex per substructure.** Interactions were divided into different categories (see Methods section for more information). Pairwise interactions established by the residues were determined using the *getcontacts* Python package. The cells were coloured according to the occupancy of the interaction (values inside the cells).


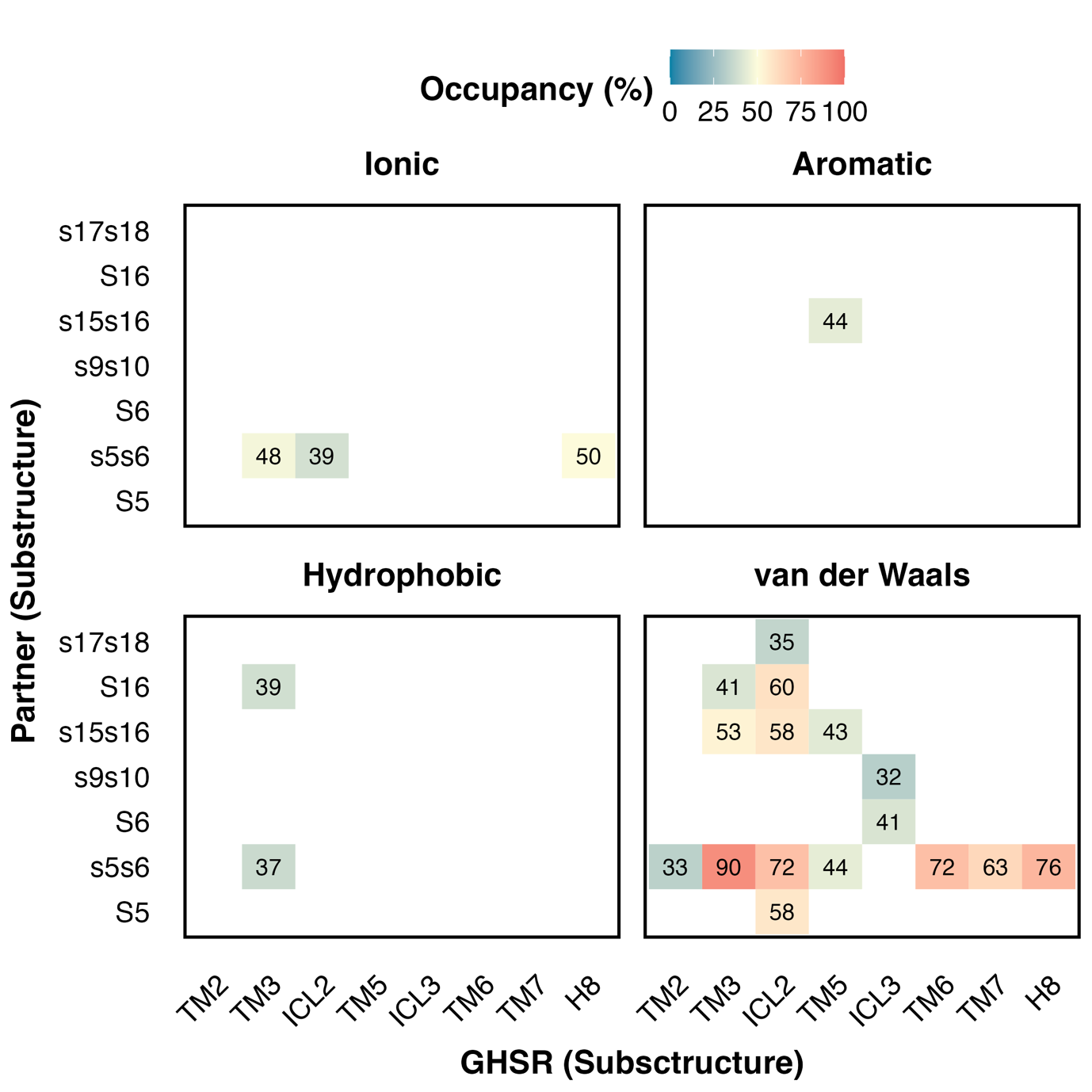


**Figure S28**: **Interaction matrix of GHSR - arr6U1N complex per substructure.** Interactions were divided into different categories (see Methods section for more information). Pairwise interactions established by the residues were determined using the *getcontacts* Python package. The cells were coloured according to the occupancy of the interaction (values inside the cells).


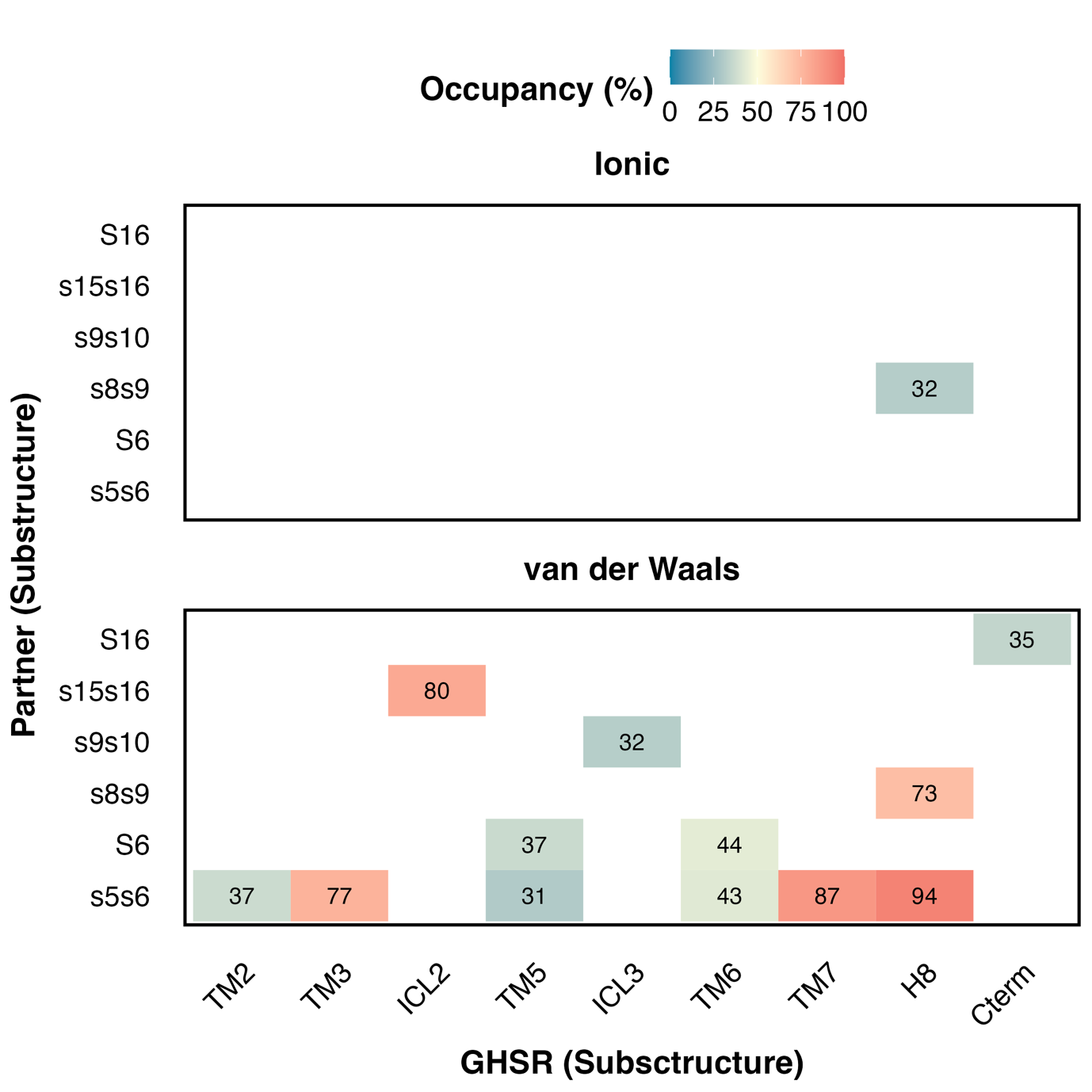


**Figure S29: Interaction matrix of the GHSR - arr6UP7 complex per substructure.** Interactions were divided into different categories (see Methods section for more information). Pairwise interactions established by the residues were determined using the *getcontacts* Python package. The cells were coloured according to the occupancy of the interaction (values inside the cells).


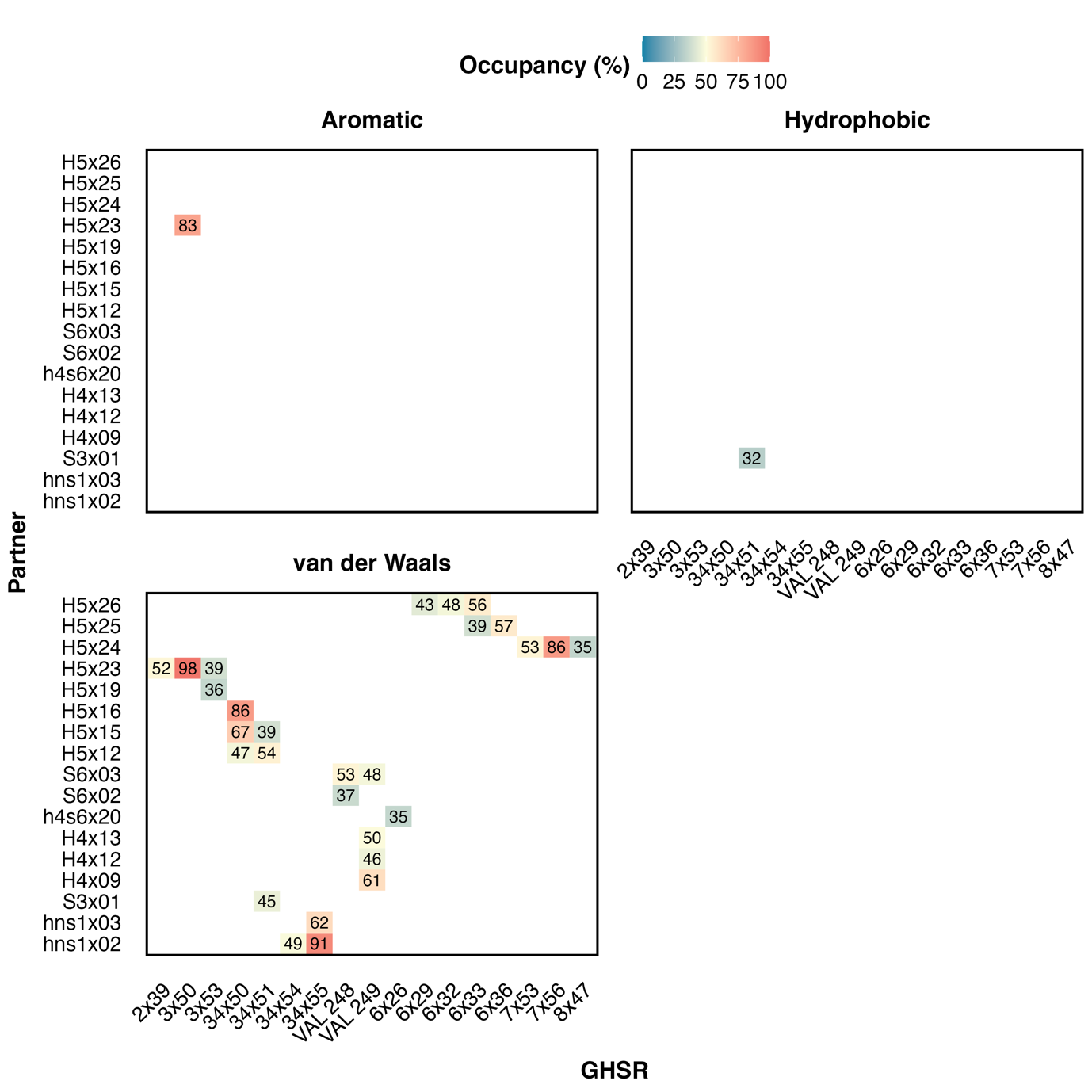


**Figure S30: Interaction matrix of the GHSR-G_q_ complex per residue.** Interactions were divided into different categories (see Methods section for more information). Pairwise interactions established by the residues were determined using the *getcontacts* Python package. The cells were coloured according to the occupancy of the interaction (values inside the cells).


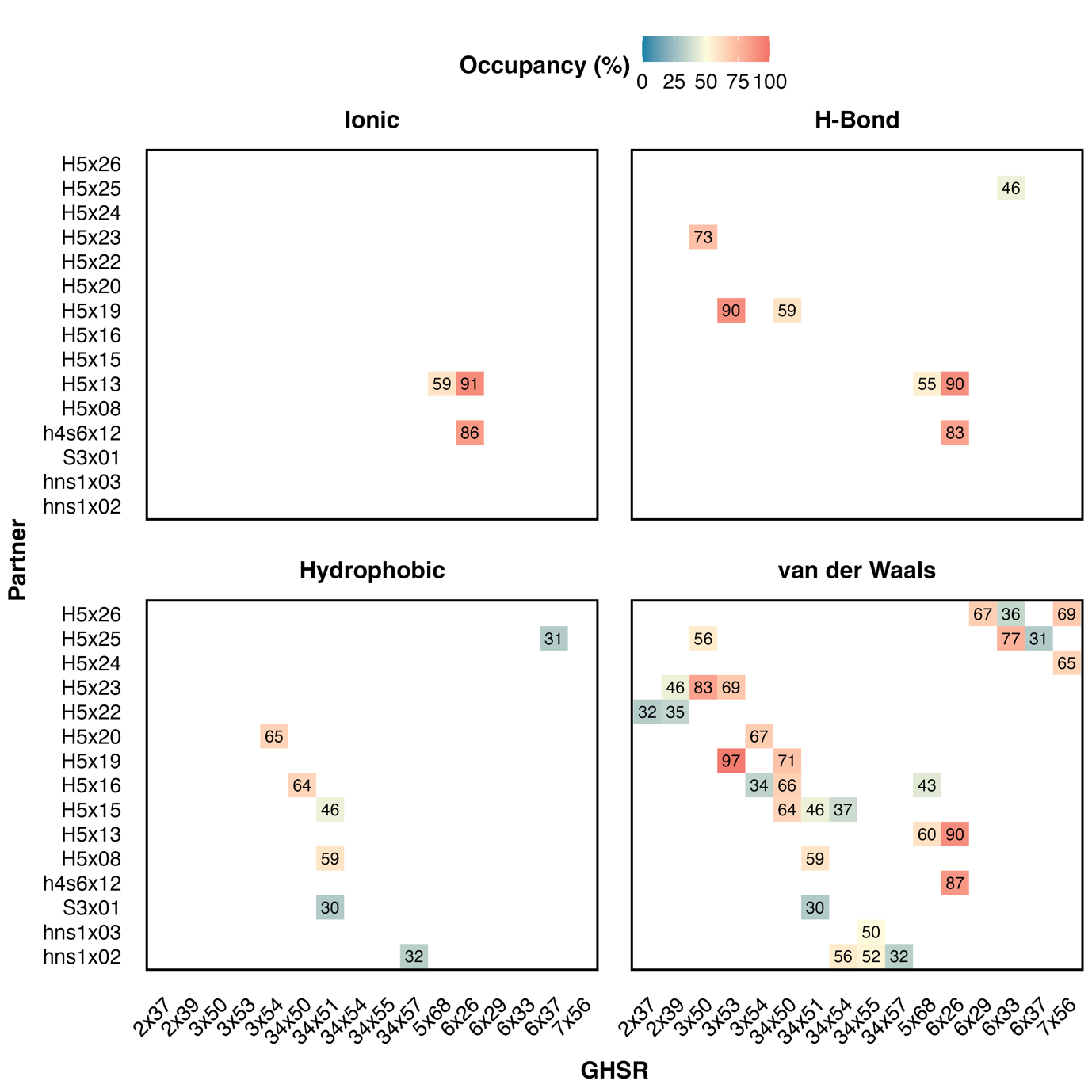


**Figure S31: Interaction matrix of the GHSR-G_i_-Ibu complex per residue.** Interactions were divided into different categories (see Methods section for more information). Pairwise interactions established by the residues were determined using the *getcontacts* Python package. The cells were coloured according to the occupancy of the interaction (values inside the cells).


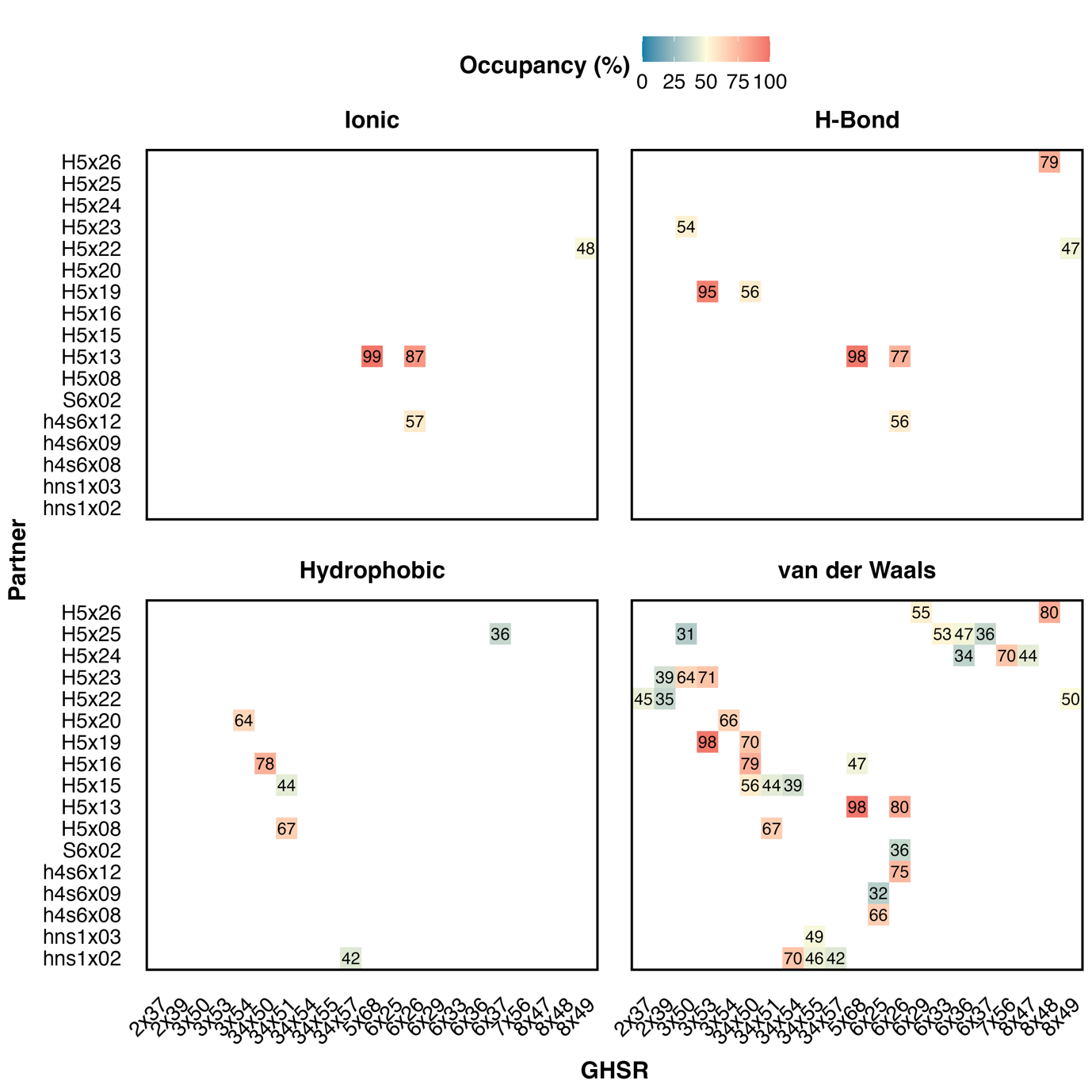


**Figure S32: Interaction matrix of the GHSR-G_i_-Ghrl complex per residue.** Interactions were divided into different categories (see Methods section for more information). Pairwise interactions established by the residues were determined using the *getcontacts* Python package. The cells were coloured according to the occupancy of the interaction (values inside the cells).


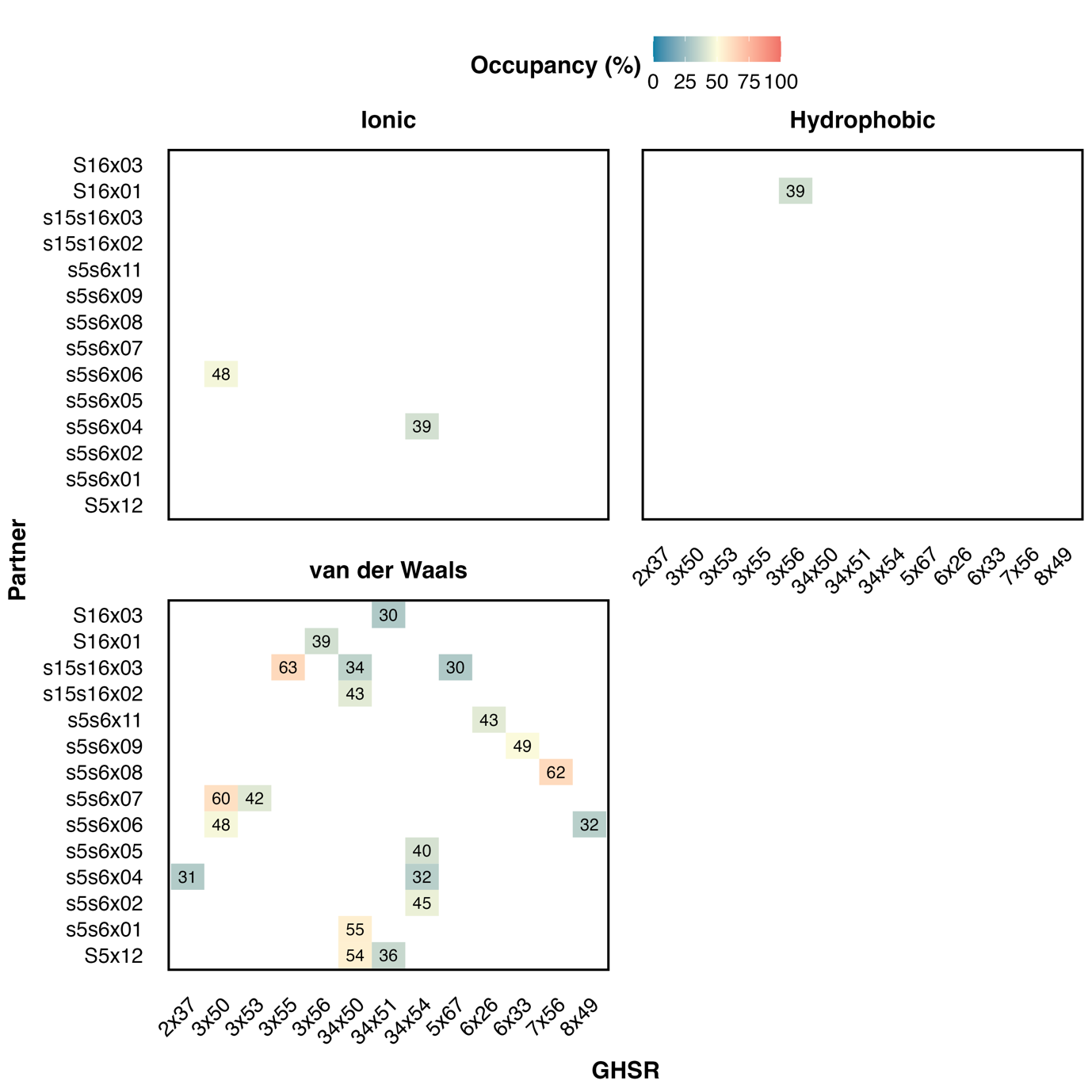


Figure S33: Interaction matrix of the GHSR - arr6U1N complex per residue. Interactions were divided into different categories (see Methods section for more information). Pairwise interactions established by the residues were determined using the *getcontacts* Python package. The cells were coloured according to the occupancy of the interaction (values inside the cells).


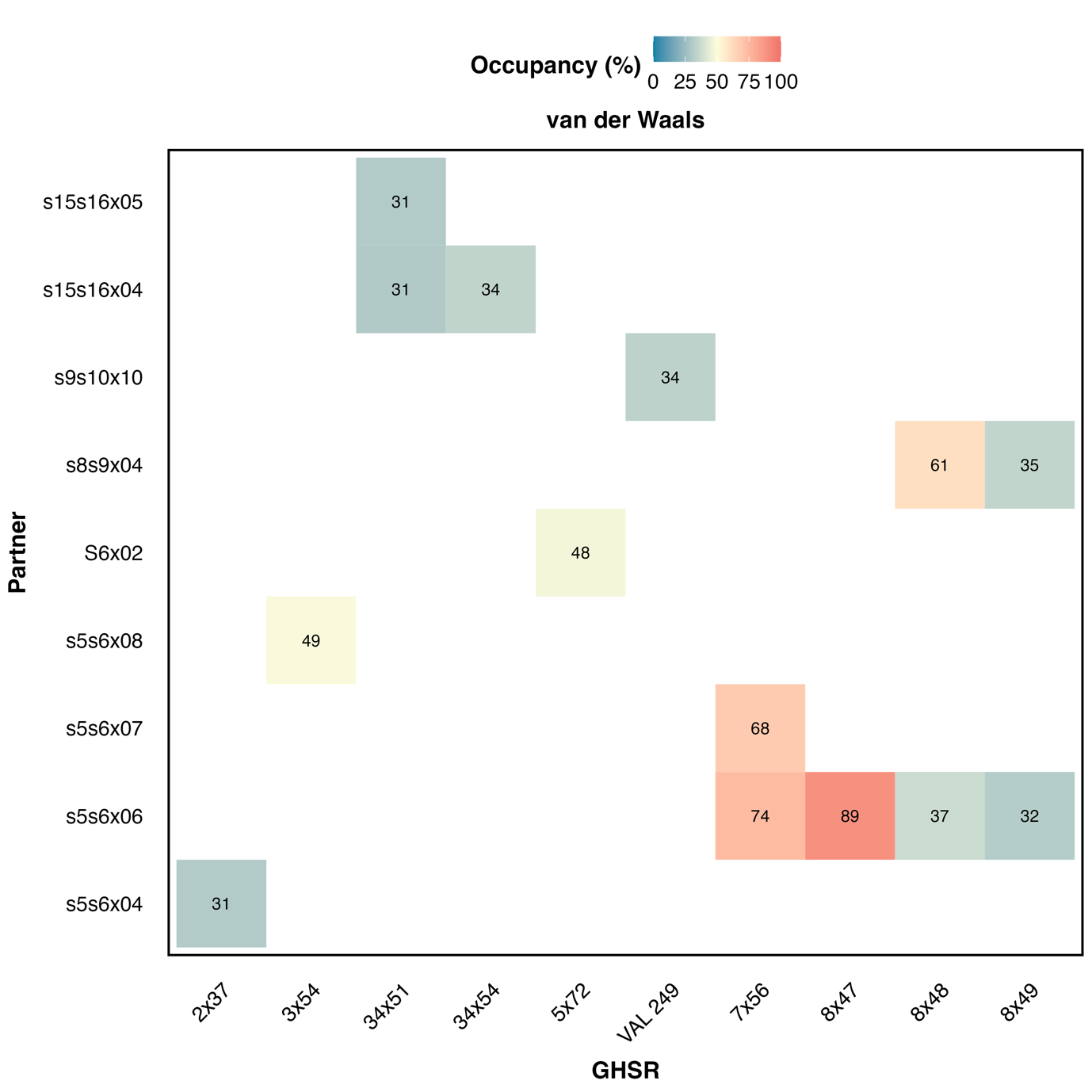


**Figure S34: Interaction matrix of the GHSR - arr6UP7 complex per residue.** Interactions were divided into different categories (see Methods section for more information). Pairwise interactions established by the residues were determined using the *getcontacts* Python package. The cells were coloured according to the occupancy of the interaction (values inside the cells).
